# Construction and Modeling of a Coculture Microplate for Real-Time Measurement of Microbial Interactions

**DOI:** 10.1128/msystems.00017-21

**Published:** 2023-02-21

**Authors:** Charles Jo, David B. Bernstein, Natalie Vaisman, Horacio M. Frydman, Daniel Segrè

**Affiliations:** a Department of Biomedical Engineering, Boston University, Boston, Massachusetts, USA; b Biological Design Center, Boston University, Boston, Massachusetts, USA; c Department of Biology, Boston University, Boston, Massachusetts, USA; d CAPES Foundation, Ministry of Education of Brazil, Brasília, Brazil; e Program in Bioinformatics, Boston University, Boston, Massachusetts, USA; f Department of Physics, Boston University, Boston, Massachusetts, USA; University of Michigan—Ann Arbor

**Keywords:** 3D printed device, 96-well plate, coculture, mathematical modeling, metabolic cross-feeding, microbial consortia, microbial interactions, microbiome, porous membrane, synthetic ecology

## Abstract

The dynamic structures of microbial communities emerge from the complex network of interactions between their constituent microorganisms. Quantitative measurements of these interactions are important for understanding and engineering ecosystem structure. Here, we present the development and application of the BioMe plate, a redesigned microplate device in which pairs of wells are separated by porous membranes. BioMe facilitates the measurement of dynamic microbial interactions and integrates easily with standard laboratory equipment. We first applied BioMe to recapitulate recently characterized, natural symbiotic interactions between bacteria isolated from the Drosophila melanogaster gut microbiome. Specifically, the BioMe plate allowed us to observe the benefit provided by two *Lactobacillus* strains to an *Acetobacter* strain. We next explored the use of BioMe to gain quantitative insight into the engineered obligate syntrophic interaction between a pair of Escherichia coli amino acid auxotrophs. We integrated experimental observations with a mechanistic computational model to quantify key parameters associated with this syntrophic interaction, including metabolite secretion and diffusion rates. This model also allowed us to explain the slow growth observed for auxotrophs growing in adjacent wells by demonstrating that, under the relevant range of parameters, local exchange between auxotrophs is essential for efficient growth. The BioMe plate provides a scalable and flexible approach for the study of dynamic microbial interactions.

**IMPORTANCE** Microbial communities participate in many essential processes from biogeochemical cycles to the maintenance of human health. The structure and functions of these communities are dynamic properties that depend on poorly understood interactions among different species. Unraveling these interactions is therefore a crucial step toward understanding natural microbiota and engineering artificial ones. Microbial interactions have been difficult to measure directly, largely due to limitations of existing methods to disentangle the contribution of different organisms in mixed cocultures. To overcome these limitations, we developed the BioMe plate, a custom microplate-based device that enables direct measurement of microbial interactions, by detecting the abundance of segregated populations of microbes that can exchange small molecules through a membrane. We demonstrated the possible application of the BioMe plate for studying both natural and artificial consortia. BioMe is a scalable and accessible platform that can be used to broadly characterize microbial interactions mediated by diffusible molecules.

## INTRODUCTION

The structures of microbial communities—their composition, diversity, and stability—are emergent properties shaped by the interactions between their constituents (1–5). There are many possible modes of interaction between microorganisms. Direct interactions, which require cell-cell contact, include mechanisms like bacterial conjugation, contact-dependent growth inhibition systems and intercellular nanotubes (6–9). Microorganisms may also interact via diffusion-mediated mechanisms, such as competition for shared nutrients, the production of toxins or communal resources, modulation of environmental conditions, quorum sensing, plasmid exchange, and metabolic cross-feeding (10–14). These multifaceted interactions within a microbial community have a profound impact on its composition, diversity, and stability (2, 4, 15–17). An appreciation for and improved understanding of microbial interactions can elucidate the metabolic and ecological principles of microbial community assembly, as well as facilitate the rational design of novel microbial consortia.

While several approaches have been developed to measure different kinds of intermicrobial interactions, the task of determining the dynamic effect of one microbe’s growth on another remains a challenging task (2, 3, 18–20). Mixed cocultures, where distinct microbes are cultured in the same vessel, are commonplace. However, measuring the abundance of individual members in a coculture is generally difficult and laborious and requires sampling for CFU counting (5) or imaging, which is usually restricted to fluorescently labeled strains (21). Plating assays are also prevalent, where a microbe is cocultured on or in proximity to a colony of another microbial species (22–24). Conditioned media assays can also be used to determine how the metabolic by-products and environmental modifications of one microbe affect the growth of another (4, 25). However, plating assays are limited by their necessity for phenotypic differentiation between microbes and a lack of convenient quantitative readouts such as optical density, whereas conditioned medium assays are restricted to the study of unidirectional, nonconcurrent interactions and would not capture the exchange of any unstable compounds. Metagenomic and 16S amplicon sequencing are increasingly used to measure relative changes in species abundance, but these approaches are ultimately limited by cost and technical challenges, including manual sampling, sample library preparation, and the introduction of additional biases (26–28).

To address these methodological limitations, systems and devices have been developed to measure cocultured microbial growth (29). High-throughput microfluidics and microdroplet based approaches have been designed to screen large numbers of multispecies microbial interactions (30–34). Although these approaches offer exciting potential, they can be difficult to apply using standard laboratory equipment, require extensive training and expertise (limiting their accessibility), and often do not allow for the simultaneous observation of the growth of each coculture member. Alternatively, coculture systems that utilize a porous membrane to physically isolate individual cultures and to allow for the exchange of diffusible molecules have been designed to enable concurrent growth and measurements of distinct microbial cultures (35–38). While these systems restrict microbial interactions to those mediated by diffusible molecules, they have shown promise for culturing previously uncultivated organisms and simultaneously measuring individual growth dynamics in microbial cocultures. One example is the iChip device, which can be used to culture previously uncultured microbes by embedding them in a natural environment while separated by a porous membrane that allows for the exchange of metabolites (36, 39). However, this device does not enable the measurement of microbial growth curves that can provide insight into the underlying interactions. Another example, the coculture plate of Moutinho et al. (35), can simultaneously measure growth curves for interacting microbes separated by a membrane in up to 8 pairs. Despite the promise of existing membrane-based systems, improvements in their throughput, ease of manufacturing, and ability to seamlessly integrate with common laboratory equipment, alongside continued application of these devices to study novel experimental systems, will enhance their use for the measurement of microbial interactions.

Here, we present the BioMe plate, a 96-well microplate-based coculture laboratory device developed to facilitate the observation of dynamic microbial interactions. BioMe separates interacting microbial cultures with a porous membrane, physically isolating individual cultures while allowing for the exchange of diffusible molecules. The growth dynamics of interacting microbial cultures can be measured independently using spectroscopic methods of standard laboratory plate readers. The BioMe plate provides a 96-well format and a three-dimensional (3D) printed design that facilitates manufacturing and modifications. The BioMe plate can be used to measure up to 30 interacting microbial pairs and is applied to two novel experimental systems.

We first used BioMe to observe a known symbiotic interaction potentially relevant to the microbiome of a host organism. We chose to study a recently reported symbiosis between organisms of the genera *Acetobacter* and *Lactobacillus* isolated from the gut of a Drosophila melanogaster laboratory stock (40). The D. melanogaster gut microbiome is relatively simple, hosting low bacterial diversity (1 to 30 species). *Acetobacter* and *Lactobacillus* are the most commonly found bacterial genera in both lab-reared and wild D. melanogaster flies, affecting development, metabolism, and behavior (41–43). The presence of these two genera has been suggested to contribute to deterministic processes related to the assembly of the D. melanogaster microbiome (44), as they interact through the exchange of metabolic waste products from *Lactobacillus* to *Acetobacter* (40). Here, we use BioMe to implement pairwise cocultures between *A. oryzifermentans* and two distinct *Lactobacillus* strains (Lactobacillus plantarum and L. brevis) isolated from lab-reared D. melanogaster stocks (45), providing additional insight into their symbiotic interactions.

We next sought to demonstrate the use of the BioMe plate to facilitate the quantitative investigation of a reduced, well-controlled microbial interaction. Specifically, we used the BioMe plate to study the syntrophic interaction between two Escherichia coli amino acid auxotrophs. Engineered auxotrophic bacteria have been used as compelling model systems for the study of microbial interactions due to their well characterized metabolic requirements (46–48). In the work of Mee et al. (46), E. coli strains were genetically recombineered for a unique single amino acid auxotrophy and then grown together in mixed cocultures to identify syntrophic pairs, where distinct amino acid auxotrophs could sustain each other’s growth. While this study demonstrated syntrophic interactions by measuring the combined optical densities (OD) of mixed cocultures, it lacked the capacity to provide additional quantitative insight into the interactions by measuring each interacting partner’s growth dynamics separately. Two E. coli auxotrophs from this study, the lysine and isoleucine auxotrophs (referred to as ΔLys and ΔIle in the text), were cocultured in the BioMe plate to further characterize and quantify their syntrophic interaction.

In parallel to the device itself, we developed a computational model of the growth and nutrient dynamics of two strains grown in connected BioMe wells. We applied this model to the syntrophic E. coli interaction to determine the factors that underlie their codependent cross-feeding relationship. In particular, we used this model, alongside an approximate Bayesian computation-based approach, to infer plausible ranges for interaction parameters related to the amino acid’s diffusion across the membrane and leakage out of E. coli cells. Together, these efforts demonstrate the scope of novel questions and experiments that are enabled by the BioMe plate, ultimately improving our understanding of the metabolic interactions and ecological relationships that shape microbial community structure.

## RESULTS

### BioMe development.

**FIG 1:**
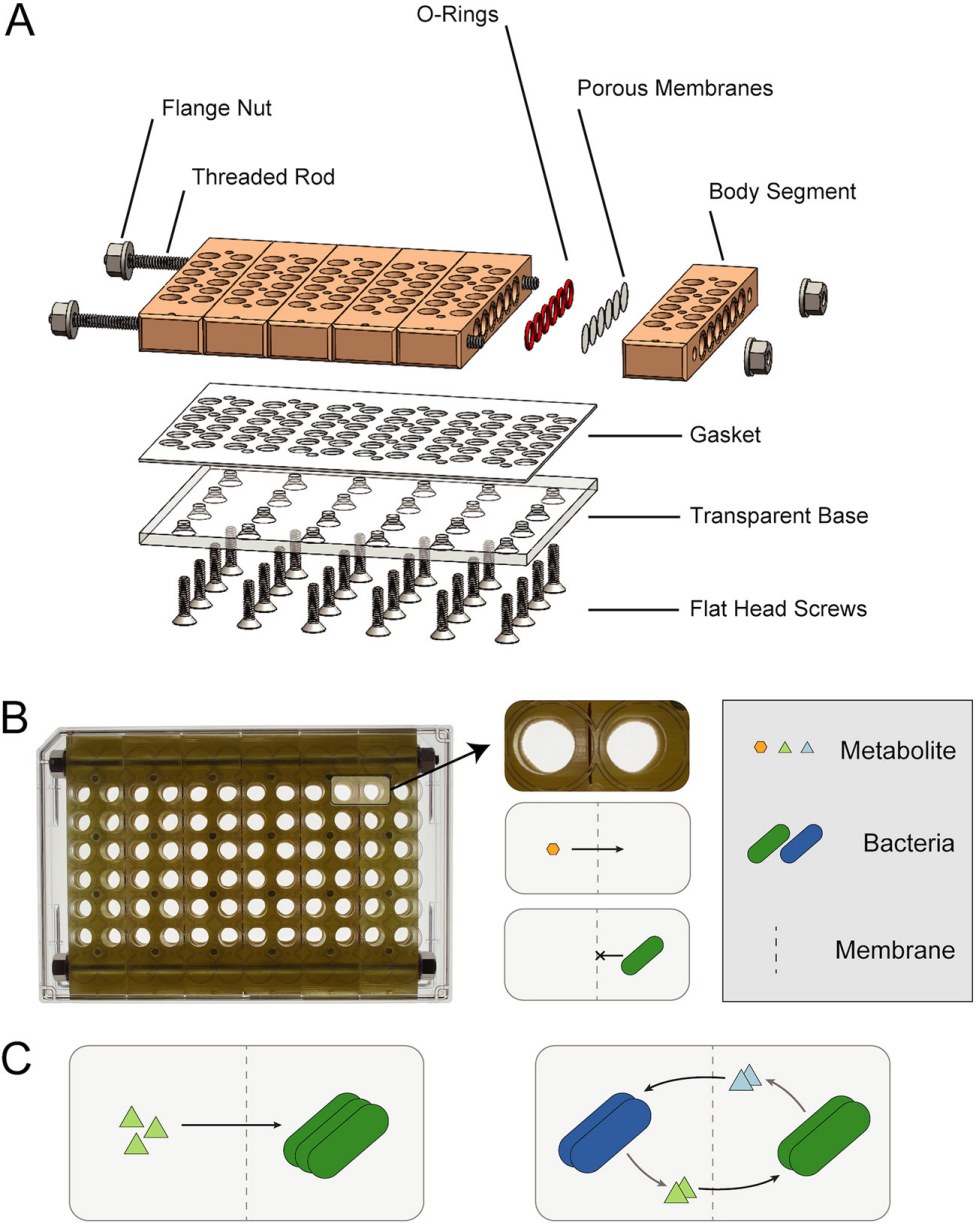
BioMe plate. (A) Exploded view of the computer-aided design (CAD) of the BioMe plate. The different components are assembled to form a microplate-based coculture device. (B) Photograph of the fully assembled BioMe plate (top view). Adjacent wells are separated by a porous membrane that allows the diffusion of metabolites and small molecules but not microbes. (C) The BioMe plate enables a variety of microbiological experiments. This includes metabolite growth assays (left) to observe microbial population growth when seeded across from a metabolite and coculture assays (right) to characterize and quantify the effects of cocultured growth between a pair of interacting microbial cultures.

We developed a microplate-based coculture device, the BioMe plate, which enables the quantitative measurement of microbial interactions (Fig. 1). A series of vertical porous membranes physically isolate constituent members of pairwise microbial interactions while allowing for the exchange of diffusible molecules. The physical segregation of interacting microbial cultures enables real-time growth dynamics measurements of each microbial population. A range of microscale pore sizes can be selected, depending on the user’s desired application, molecule size selectivity, and degree of diffusion across the membrane.

The BioMe plate is comprised of several components, which are further detailed in Table S1 in the supplemental material. The standard 96-well microplate design is fragmented into six discrete body segments, which form the wells of the device. Each well has an opening on the side of the body segment that connects it to its respective coculture well in a separate body segment, via O-ring and porous polycarbonate membrane. The body segments are laterally fastened and sealed using rods and nuts, yielding a total capacity of 30 pairwise coculture assays. Of note, the body segments can be machined from polypropylene or stereolithography 3D printed using a biocompatible and autoclavable dental resin, circumventing the need for labor-intensive machining. A transparent base forms the bottom of the plate, allowing for real-time spectroscopic readings (OD or fluorescence) of each well. The base is machined from polycarbonate sheet and is vertically fastened and sealed to the assembled body chassis via a laser-cut gasket and screws. This forms the core of the BioMe plate, which is housed in between two clear 96-well plate lids sealed with Parafilm to prevent evaporation and equipment damage if leakage were to ever occur. No significant evaporation was observed under this set up for experiments run for up to 96 h.

10.1128/mSystems.00017-21.10 TABLE S1 BioMe materials. A list of all components for construction of the BioMe plate, including vendor and catalogue number, is presented. Download Table S1, XLSX file, 0.01 MB. Copyright © 2023 Jo et al. 2023 Jo et al. https://creativecommons.org/licenses/by/4.0/ This content is distributed under the terms of the Creative Commons Attribution 4.0 International license.

### BioMe is sterilizable and leakproof and enables small molecule diffusion.

A sterilized BioMe plate can be reused to ensure cost-effective, contamination-free repeated use. A single plate was sterilized and reused for all the experiments in this work, with no detectable deterioration in performance. The porous membranes are disposable and replaced per use. Leakage tests demonstrated a water-tight seal throughout (see “Leakage test” in Materials and Methods) and sterilization validation tests demonstrated successful decontamination of the BioMe plate (see “Sterilization protocol and validation” in Materials and Methods). The ability of various membrane pore sizes to restrict crossover of microbial cells was tested; crossover was observed in several experiments, especially for larger pore sizes (0.4 μm) and rarely for smaller pore sizes (0.03, 0.1, and 0.2 μm [see “Syntrophic E. coli amino acid auxotroph interaction” in Results; see also Discussion]).

**FIG 2:**
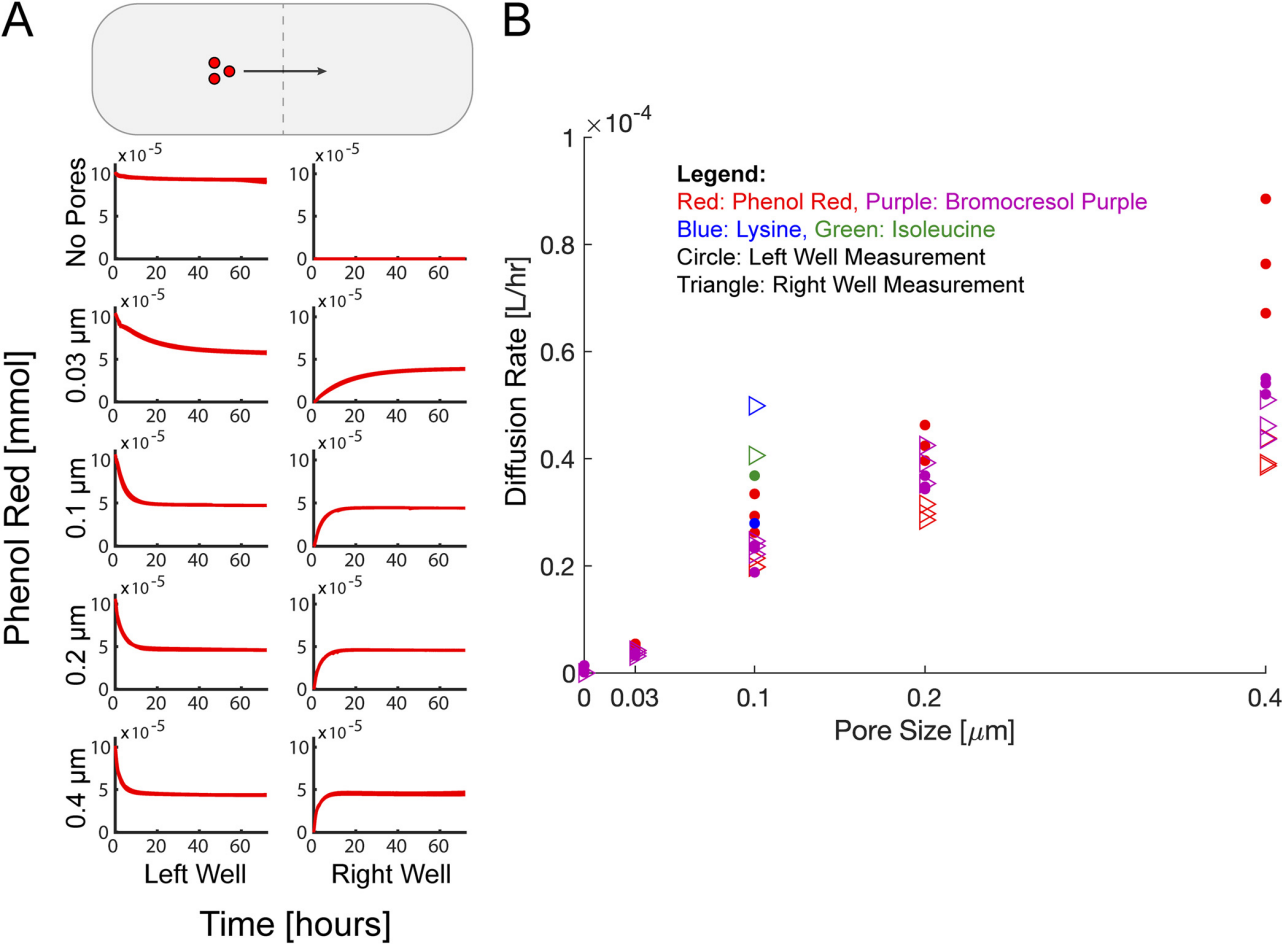
Chemical dye and amino acid diffusion across membranes with variable pore size in the BioMe plate. A small-molecule dye (400 μM concentration of phenol red or bromocresol purple) was loaded into columns of wells to the left of the membrane in the BioMe plate, for varying membrane pore sizes: no pores and 0.03-, 0.1-, 0.2-, and 0.4-μm pores. The OD at the isosbestic point (478 nm for phenol red, 490 nm for bromocresol purple) was measured for 72 h at 15-min intervals. The dye concentration was inferred by linear calibration to optical density. The amino acid diffusion was similarly measured for 400 μM concentrations of lysine and isoleucine (see “Amino acid diffusion measurement” in Materials and Methods) (A) Time course of calibrated dye concentration for phenol red with membranes of different pore sizes. Replicates (*n* = 3; all overlapping curves are shown on the plot) were conducted for each pore size. (B) Diffusion rates for phenol red and bromocresol purple for various membrane pore sizes and for lysine and isoleucine for the 0.1-μm pore size. Diffusion rates were calculated by fitting the measured concentration time course to the diffusion model using data from both the left and right well measurements.

Small molecule diffusion across the porous membranes was tested using colorimetric assay dyes. Specifically, the BioMe plate was loaded with two different dyes, phenol red (354.38 g/mol) and bromocresol purple (540.24 g/mol), and their diffusion across membranes with various pore sizes was monitored (Fig. 2A; see also Fig. S1). The membranes are hydrophilic polycarbonate disks with variously sized pores created using track etching. Phenol red and bromocresol purple are typically used as pH indicators, but when measured at their isosbestic points (478 and 490 nm, respectively) (49, 50), their concentrations can be inferred from optical density (absorbance measurements). We developed calibration curves for each dye to ensure accurate extrapolation of concentration from optical density (see Fig. S2). Both dyes were observed to diffuse across the porous membranes with a diffusion rate that increased monotonically for increasing pore size and were not observed to diffuse across membranes with no pores (the same material as the membranes, polycarbonate discs, with no etched pores). The raw OD data for the diffusion and calibration experiments, laid out as they were on the BioMe plate, are shown in Fig. S9 and S10 (https://github.com/segrelab/co_culture_device). In addition, we experimentally measured the diffusion of the amino acids lysine and isoleucine across the 0.1-μm-pore-size membrane in the BioMe device (see Fig. S3; see also “Amino acid diffusion measurement” in Materials and Methods). This measurement confirmed the diffusion of these amino acids in our device and provided quantitative information for our computational modeling work.

10.1128/mSystems.00017-21.2 FIG S1 Bromocresol purple diffusion across the BioMe plate. Small-molecule dye (bromocresol purple) was placed on one side of the membrane in the BioMe plate for membranes of different pore sizes. Three replicates are shown here (overlapping) for each pore size. The amount of dye was measured by optical density at the isosbestic point (optical density was converted to amount by a linear calibration shown in Fig. S2). Download FIG S1, JPG file, 1.0 MB. Copyright © 2023 Jo et al. 2023 Jo et al. https://creativecommons.org/licenses/by/4.0/ This content is distributed under the terms of the Creative Commons Attribution 4.0 International license.

10.1128/mSystems.00017-21.3 FIG S2 Small molecule dye calibration curves. (A and B) Linear calibration converting phenol red (A) and bromocresol purple (B) OD at the isosbestic point to concentration. Download FIG S2, JPG file, 0.8 MB. Copyright © 2023 Jo et al. 2023 Jo et al. https://creativecommons.org/licenses/by/4.0/ This content is distributed under the terms of the Creative Commons Attribution 4.0 International license.

10.1128/mSystems.00017-21.4 FIG S3 Amino acid calibration and diffusion curves. Lysine and isoleucine were diffused across the BioMe device with a 0.1-μm-pore-size membrane. (A) Calibration curves relating enzyme assay measurements to amino acid concentrations. (B) Amino acid concentration curves for the left well (blue), the right well (red), and the total (black). Dotted lines show average concentration time courses across all three replicates. Download FIG S3, JPG file, 1.8 MB. Copyright © 2023 Jo et al. 2023 Jo et al. https://creativecommons.org/licenses/by/4.0/ This content is distributed under the terms of the Creative Commons Attribution 4.0 International license.

Diffusion rates of all tested membrane pore sizes for each chemical dye, and amino acids, were inferred using a gradient-driven diffusion model (further described in the “Computational modeling” sections below). This model gives rise to an exponential function describing the time-dependent concentration of the molecules in either well of the system, as the metabolites diffuse across the membrane (see Text S1). An exponential function was fit to the diffusion data (see Materials and Methods; see also Fig. S11 [https://github.com/segrelab/co_culture_device]) to infer a range of estimates for our model’s diffusion rate. As shown in Fig. 2B, the mean diffusion rate increases monotonically and nonlinearly with increasing pore size. Interestingly, the estimated diffusion rate was consistently higher when calculated using the curves from the well where the dye was initially placed as opposed to when using the curves from the opposite well into which the dye diffused. This effect was more pronounced for the larger pore sizes and for phenol red (see Discussion for additional details). The amino acid diffusion rates for the 0.1-μm-pore-size membrane are slightly higher than the dye diffusion rates, which may be due to the smaller molecular weight of the amino acids relative to the dyes.

10.1128/mSystems.00017-21.1 TEXT S1 Equations. (A) Metabolite diffusion dynamics. (B) Computational model of E. coli amino acid auxotroph interaction. (C) Parameters and initial conditions. (D) Statistic used for approximate Bayesian computation. Download Text S1, DOCX file, 0.09 MB. Copyright © 2023 Jo et al. 2023 Jo et al. https://creativecommons.org/licenses/by/4.0/ This content is distributed under the terms of the Creative Commons Attribution 4.0 International license.

### Symbiotic interaction between *Drosophila melanogaster* gut microbiome species.

**FIG 3:**
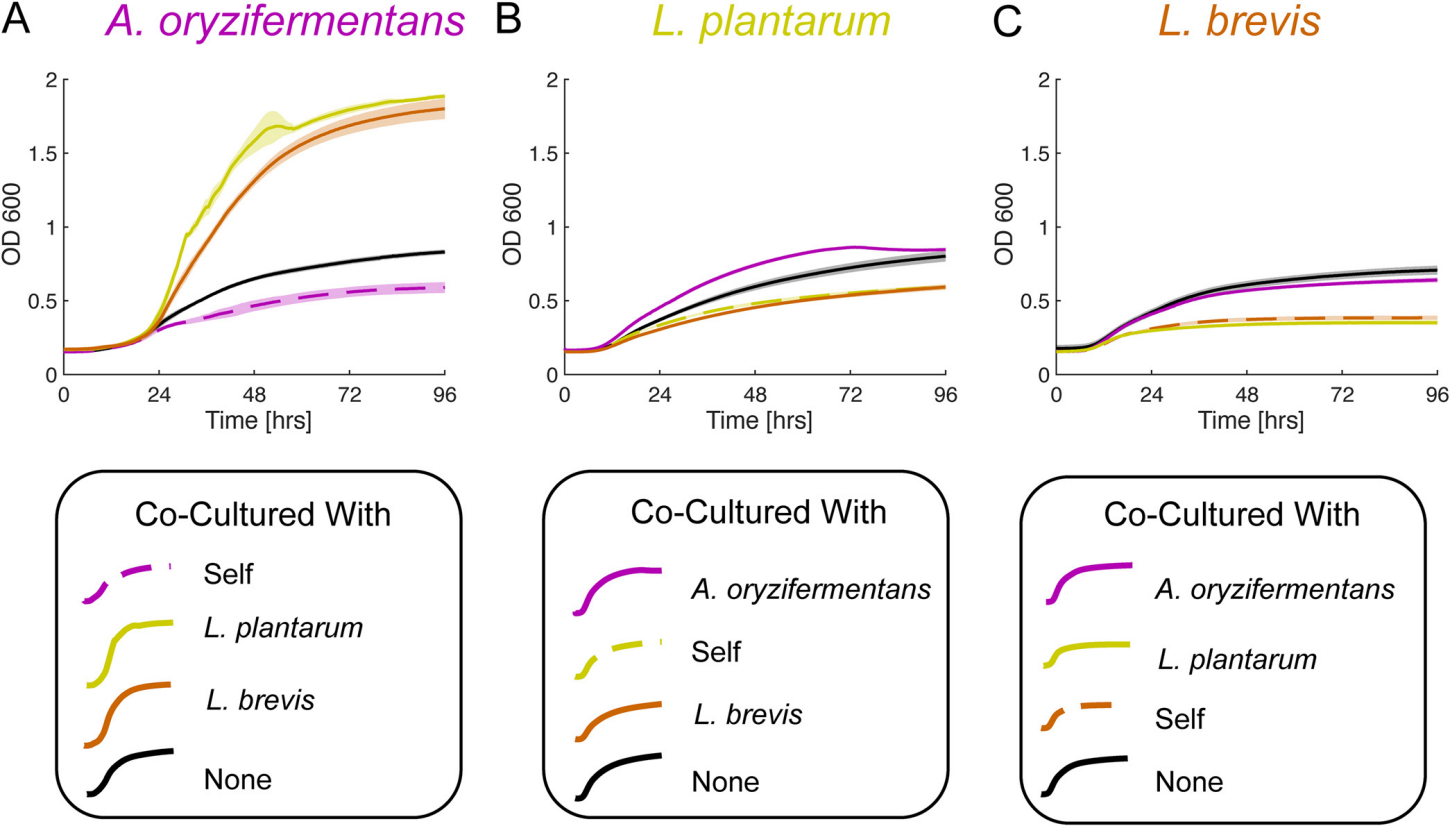
Commensal relationship between D. melanogaster gut-associated microbes. (A to C) Pairwise cocultures between all combinations of A. oryzifermentans (A), L. plantarum (B), and L. brevis (C). A membrane pore size of 0.03 μm was used throughout all of these experiments. Lines indicate mean growth curves, and shaded regions indicate standard errors (*n* = 4 for cross-species cocultures, *n* = 3 for medium-control “none,” *n* = 6 for self-control “self” for *L. plantarum* and L. brevis, and *n* = 4 for “self” for *A. oryzifermentans* cultures, as two contaminated outliers were left out, see Fig. S4; see also Fig. S12 [https://github.com/segrelab/co_culture_device] for additional details).

As a first biological application of the BioMe plate, we confirmed a recently characterized interaction among specific genera of bacteria from the D. melanogaster gut microbiome. In particular, we focused on three distinct bacterial strains previously isolated from laboratory-bred flies: one species of *Acetobacter* (A. oryzifermentans) and two species of *Lactobacillus* (L. plantarum and L. brevis) (45). The BioMe plate was used to test and observe every pairwise coculture combination between these three strains. In addition, relevant controls were included in the experiments, i.e., organisms paired with themselves (self-control) and organisms paired with wells containing growth medium but no other organism (medium-control) (Fig. 3; see also Fig. S4 and Fig. S12 [https://github.com/segrelab/co_culture_device]). A 0.03-μm-pore-size membrane was chosen to allow exchange of metabolites but ensure no microbial crossover, as done for the iChip device (39).

10.1128/mSystems.00017-21.5 FIG S4 *Drosophila* gut microbe interactions: all data and replicates. Growth curves and replicates for all coculture experiments between A. oryzifermentans (purple), L. plantarum (yellow), and L. brevis (orange) are shown. The schematic demonstrates the type of experiment performed. (A) Coculture, (B) self-interaction (self-control), (C) empty well interaction (medium-control). Download FIG S4, JPG file, 2.7 MB. Copyright © 2023 Jo et al. 2023 Jo et al. https://creativecommons.org/licenses/by/4.0/ This content is distributed under the terms of the Creative Commons Attribution 4.0 International license.

The coculture experiments revealed an increased growth of *A. oryzifermentans* when cocultured with either *Lactobacillus* strain, demonstrating a clear benefit relative to its growth when cocultured with itself or no microbe in the opposing well. Interestingly, for both L. brevis and *L. plantarum*, their growth in coculture with the *Acetobacter* strain was similar to their growth in respective medium-controls but still demonstrated improved growth relative to both their self-controls and coculture with the other *Lactobacillus* strain. Our results, uniquely enabled by the BioMe plate, suggest that the *Acetobacter* population disproportionately benefits from this metabolite-mediated interaction. BioMe could serve as the starting point for further exploration of this cross-species interaction, complementing existing approaches (40, 51, 52).

### Syntrophic interaction between *Escherichia coli* amino acid auxotrophs.

Next, we used BioMe to enable a more in-depth and quantitative study of a model microbial interaction in which two strains have been engineered to exchange essential metabolites in order to grow. Specifically, we studied the syntrophic interaction between a pair of E. coli amino acid auxotrophs: ΔLys and ΔIle. These strains were engineered to be unable to biosynthesize lysine or isoleucine, respectively, requiring supplementation of the missing amino acid to grow in monoculture. Despite their inability to grow in monoculture without supplementation, both strains are able to grow when placed together in a mixed coculture (46).

**FIG 4:**
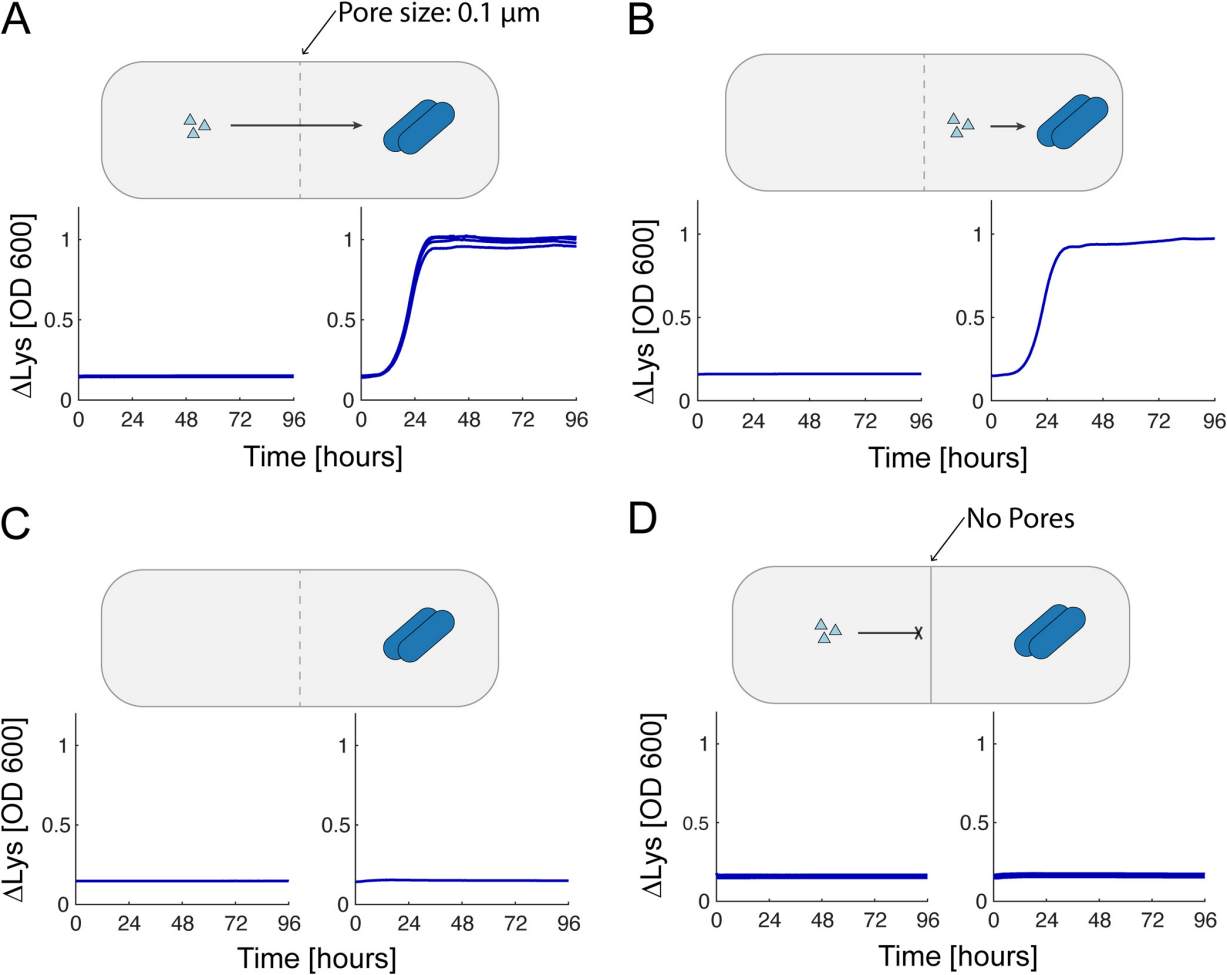
Lysine diffusion bioassay with E. coli *ΔLys* in the BioMe plate. Various amino acid diffusion conditions were tested in the BioMe plate. (A) ΔLys E. coli auxotrophs grown in minimal media with supplemental lysine across the 0.1 μm membrane (*n* = 4). (B) Positive control with lysine supplemented in the same well as the microbial culture (*n* = 1). (C) Negative control with no supplemental lysine provided (*n* = 1). (D) Negative control with supplemental lysine provided across a membrane with no pores (*n* = 4). For all minimal media supplemented with amino acids, the amino acid concentration was chosen to theoretically yield 10^9^ cells (see Materials and Methods; lysine, 0.731 mmol/L).

The first test of this system in the BioMe device was a validation that amino acids could diffuse across membranes of various pore sizes and support the growth of an auxotrophic E. coli strain in the adjacent well. Figure 4 shows an example of this validation for lysine diffusion across the 0.1-μm-pore-size membrane to support the growth of the ΔLys strain. Lysine was shown to diffuse across the membrane, with comparable growth to the positive control where lysine was added into the same well as the microbial culture (Fig. 4A and B). A negative-control experiment, lacking the supplemental amino acid, displayed no growth and thus confirmed the amino acid auxotrophy (Fig. 4C). A second negative-control experiment, displaying lack of growth for membranes with no pores, confirmed the integrity of the device seal (Fig. 4D). These results were repeated with ΔIle and isoleucine, and for various pore sizes: no pores and 0.03-, 0.1-, 0.2-, and 0.4 μm pores. We found similar results in all cases, with comparable growth between diffusion and positive-control conditions and no growth in either of the negative controls (see Fig. S5, as well as Fig. S13 and S14 [https://github.com/segrelab/co_culture_device]).

10.1128/mSystems.00017-21.6 FIG S5 E. coli auxotroph amino acid diffusion experiments - all data and replicates. The growth results for of ΔLys (A) and ΔIle (B) are shown for five different pore sizes (no pores and 0.03-, 0.1-, 0.2-, and 0.4-μm pores) alongside positive- and negative-control growth curves. Blue, ΔLys; green, ΔIle; red, negative controls; gray, positive controls. For all minimal media supplemented with lysine or isoleucine, the amino acid concentration was 0.731 or 0.498 mmol/L, respectively. Download FIG S5, JPG file, 2.8 MB. Copyright © 2023 Jo et al. 2023 Jo et al. https://creativecommons.org/licenses/by/4.0/ This content is distributed under the terms of the Creative Commons Attribution 4.0 International license.

**FIG 5:**
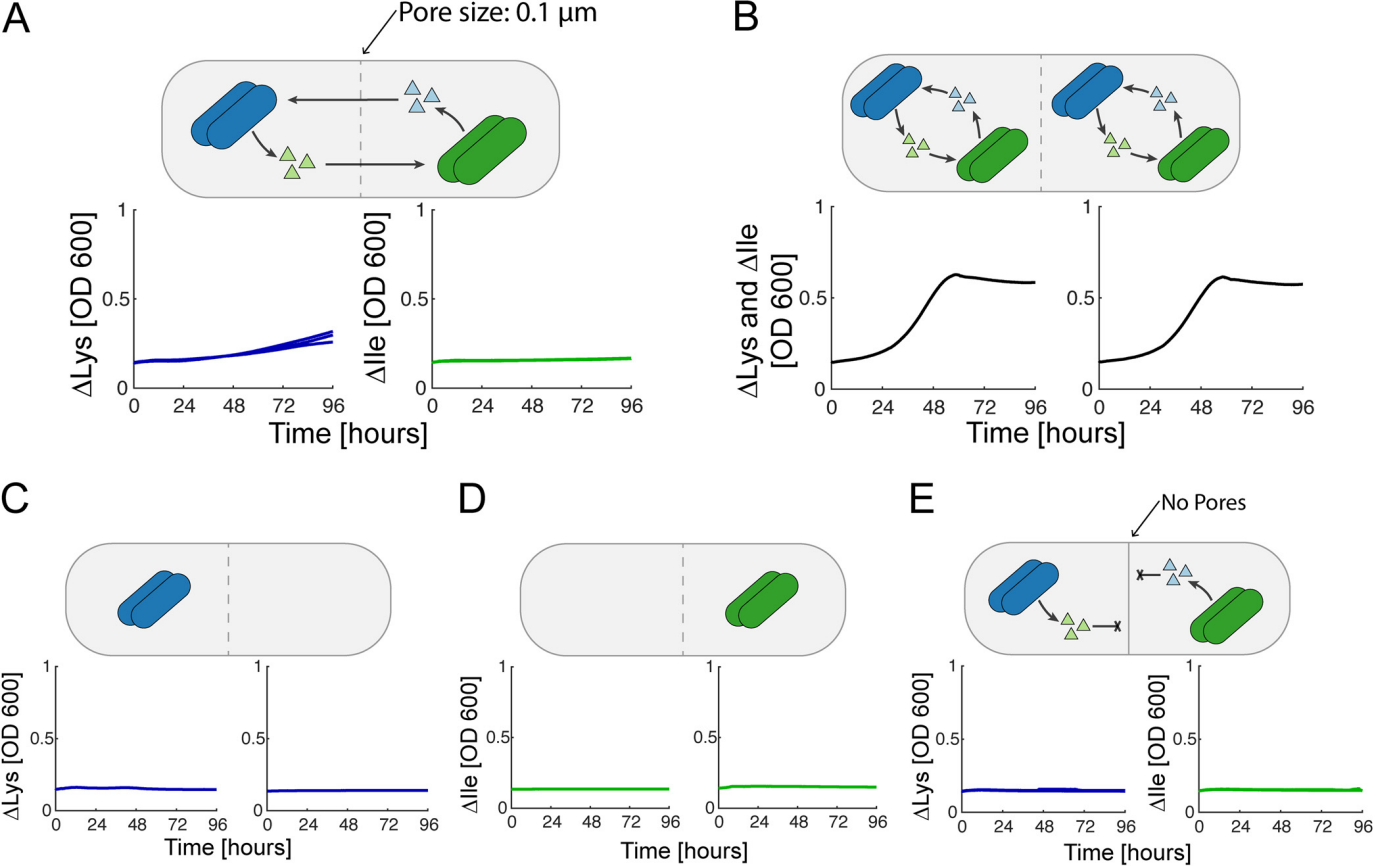
Syntrophic E. coli lysine and isoleucine auxotroph coculture. Mutualistic cross-feeding between ΔLys and ΔIle amino acid auxotrophs. (A) ΔLys (blue) and ΔIle (green) grown across a 0.1-μm porous membrane (*n* = 3). (B) Positive control with ΔLys and ΔIle grown in the same wells (*n* = 1). (C and D) Negative controls with ΔLys and ΔIle without a syntrophic partner, respectively (*n* = 1). (E) Negative control with ΔLys and ΔIle separated by a membrane with no pores (*n* = 3).

We next cocultured ΔLys and ΔIle in the BioMe plate to measure the dynamics of their metabolic interaction (Fig. 5). As expected, each strain can complement, to some extent, the amino acid missing in the other, confirming the existence of cross-feeding. However, the auxotrophs grew significantly slower when inoculated in paired wells separated by a porous membrane than when grown in the same well (Fig. 5A and B). The membrane significantly impeded the growth of both members in the syntrophic interaction. As shown in Fig. 5A, this effect is asymmetric, in that ΔIle seems to be able to help ΔLys more than ΔLys helps ΔIle, though the growth of ΔIle is still greater than that of the negative control. Negative controls further confirmed that neither auxotrophs could grow without the partner strain (Fig. 5C and D) or when separated by a membrane with no pores (Fig. 5E). Qualitatively similar results were observed for membrane pore sizes of 0.03, 0.1, and 0.2 μm (see Fig. S6; see also Fig. S16 [https://github.com/segrelab/co_culture_device]). For membranes with a 0.4-μm pore size, microbial crossover through and/or around the membrane/O-ring junction occurred in all replicates (measured by selective plating at the end of the experiment; see Fig. S7), preventing any measurement of interaction dynamics. The slowdown of cross-feeding across the membrane (Fig. 5) pointed to a concentration-dependent effect, and we wondered whether the quantitative nature of the BioMe experiments could help us gain a deeper understanding of the dynamics underpinning this phenomenon.

10.1128/mSystems.00017-21.7 FIG S6 E. coli auxotroph coculture experiments: all data and replicates. The growth curves of ΔLys and ΔIle cocultures are shown for 5 different pore sizes (No Pores, 0.03, 0.1, 0.2, and 0.4 μm) alongside positive and negative controls. Dotted lines indicate crossovers of auxotrophs as indicated in Fig. S7. Blue, ΔLys; green, ΔIle; red, negative controls; gray, positive controls. Download FIG S6, JPG file, 1.1 MB. Copyright © 2023 Jo et al. 2023 Jo et al. https://creativecommons.org/licenses/by/4.0/ This content is distributed under the terms of the Creative Commons Attribution 4.0 International license.

10.1128/mSystems.00017-21.8 FIG S7 Crossover assay. A selective platting assay at the end of the E. coli auxotroph coculture experiment was performed for all wells to identify when organisms had crossed into the adjacent well of the BioMe plate. A 5-μL aliquot from each well was cultured on minimal media agar plates supplemented with either lysine (0.731 mmol/L) or isoleucine (0.498 mmol/L). Plates were cultured for 72 h at 30°C in a static incubator. Black letters indicate that the correct auxotroph was identified in that well, and red letters indicate that the incorrect auxotroph was found in the well. Download FIG S7, JPG file, 2.4 MB. Copyright © 2023 Jo et al. 2023 Jo et al. https://creativecommons.org/licenses/by/4.0/ This content is distributed under the terms of the Creative Commons Attribution 4.0 International license.

### Computational modeling of BioMe cocultures provides insight into interaction parameters.

**FIG 6:**
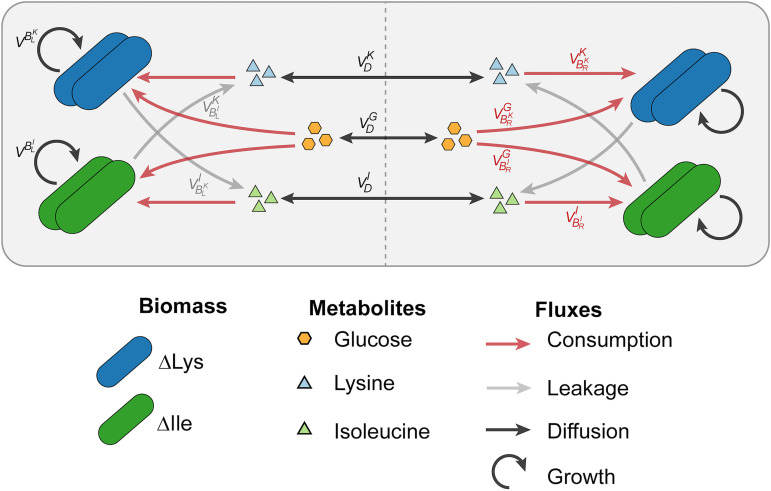
Computational model of syntrophic E. coli auxotroph growth dynamics in BioMe. The fluxes of metabolites simulated by the model are illustrated in this figure, including the diffusion of glucose and amino acids across the porous membrane (black arrows), the uptake of glucose and auxotrophy amino acids supporting growth (red arrows), and the leakage of the respective partner’s auxotrophy amino acids (gray arrows). The growth of each auxotroph’s biomass is also represented (circular arrow). Text S1 describes all parameters and the full dynamics of the system.

To further investigate the interaction dynamics observed experimentally with the BioMe plate, we developed a computational model describing the syntrophic E. coli auxotroph coculture experiments. This model captures the dynamics within two interacting wells of the BioMe plate, by simulating the processes of metabolite diffusion across the membrane, glucose and amino acid uptake by the E. coli auxotrophs, and a stoichiometric leakage of the respective partner’s auxotrophy amino acids (Fig. 6); the full list of equations and parameters is available (see Text S1; see also https://github.com/segrelab/co_culture_device). Using this computational model, all performed experiments involving the E. coli amino acid auxotrophs can be simulated by changing the initial conditions of the model to match those of the experiment (see Fig. S17 [https://github.com/segrelab/co_culture_device]).

**FIG 7:**
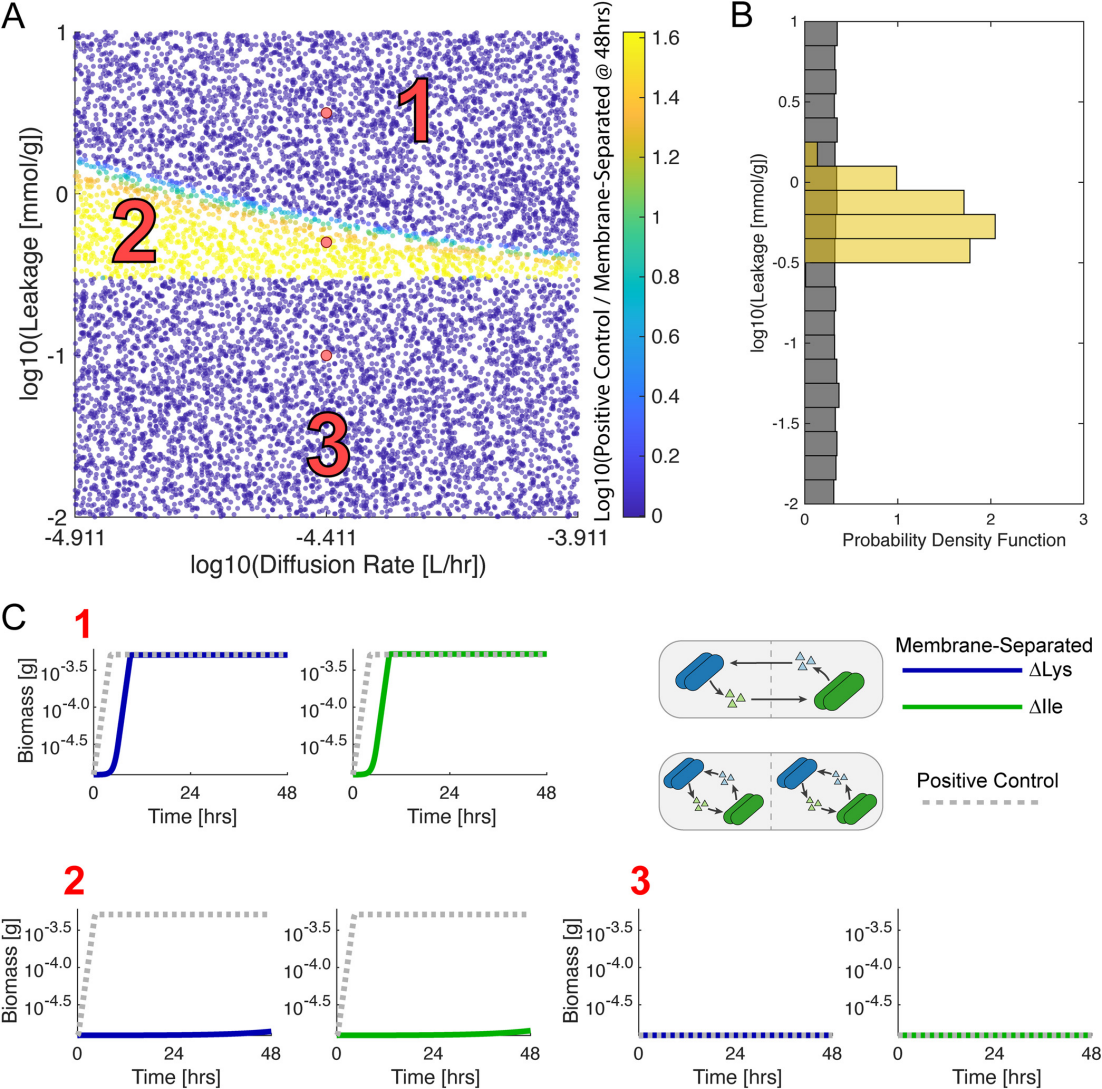
Computational modeling of E. coli auxotroph coculture interaction parameters. (A) The sampled space of diffusion and leakage parameters is shown. The diffusion rate was sampled from an order of magnitude (10×) log uniform prior range centered around 3.88 × 10^−5^ L/h (the experimentally measured mean diffusion rate of lysine and isoleucine in our experiments; see Fig. 2). The leakage rate was sampled from a 3-orders-of-magnitude (1,000×) log uniform prior range. Each point represents a different simulation of the model. The color indicates the log base 10 of the ratio between the yield at 48 h of E. coli auxotrophs grown in the same well (positive control) divided by the yield in opposite wells (membrane-separated). Sampled points within 2× the standard deviation (0.235) of the mean (1.14) of this statistic were used to sample the posterior distribution in approximate Bayesian computation. (B) The prior (gray) and posterior (gold) distributions of the leakage stoichiometry parameter are plotted as histograms. The prior distribution was sampled from a log uniform distribution. (C) Example growth curves for different regions of the parameter space in panel A are shown. Simulated growth curves of ΔLys (blue line) and ΔIle (green line) grown across from each other (membrane-separated) and grown together (gray dotted line, positive control) are shown for three different regions of parameter space.

We integrated this computational model with our syntrophic coculture experiment to explore the parameter space that gives rise to our primary qualitative observation, i.e., that both members of the mutualistically cross-feeding pair grew significantly faster when in the same well than when interacting across the membrane. For this modeling work, we used the data from the 0.1-μm-pore-size experiment since there was no microbial crossover in any of the three replicates for this set of cocultures, although the results are qualitatively similar in the 0.03- and 0.2-μm-pore-size cocultures. The parameters for uptake kinetics and biomass stoichiometry were estimated from the literature (see Text S1). The parameter space was then explored for two important parameters of the interaction, which were difficult to estimate from the literature: the diffusion rate of the metabolites across the membrane and the leakage stoichiometry of the amino acids. The diffusion rate was informed by experimental measurements of lysine and isoleucine diffusion across a 0.1-μm-pore-size membrane (see Fig. S3), while the leakage stoichiometry was sampled from a large range of possible values. Upon varying these parameters, we compared the 48-h predicted growth yield of an auxotroph grown in the same well with its partner relative to the yield in membrane-separated coculture (Fig. 7A). Distinct regions were clearly visible in this parameter space, which gave rise to different qualitative simulated results (Fig. 7A and C). One can see that if the cellular leakage of the amino acid is too small (Fig. 7A, region 3), the same-well and separate-well simulations behave similarly, i.e., neither supports any growth. If both leakage and diffusion are high enough (Fig. 7A, region 1), the two *in silico* experiments again behave similarly, but this time they both lead to an increased yield. Notably, there is an intermediate region of the parameter space (Fig. 7A, region 2) in which leakage is not a limiting step to guarantee cross-feeding, but diffusion between separate wells is. In this region, the two auxotrophs can secrete enough amino acids to support each other, but diffusion through the BioMe membrane slows down the growth process enough to lead to a detectable difference.

The identification of the intermediate region (Fig. 7A, region 2) prompted us explore the possibility of using this modeling framework to provide more insight into the experimentally unknown key parameters. We used an approximate Bayesian computation approach (see Materials and Methods) to infer—and model the uncertainty of—the leakage parameter values that were consistent with the observed differential growth yields in the same-well positive control versus the membrane-separated coculture (Fig. 7B). The posterior distribution of the leakage parameter, conditioned on this experimental observation, was estimated using a statistic of the logarithm of the ratio of the biomass yields at 48 h (see Text S1), and an acceptance cut off centered around the experimentally measured mean value (see Materials and Methods for further details). To further investigate the asymmetric nature of our observations, a similar analysis was conducted where the lysine and isoleucine leakage parameters were sampled from independent prior distributions, and could thus be inferred separately (see Fig. S8). This gave rise to a lysine leakage parameter that was higher than that of isoleucine, consistent with our observation of increased ΔLys growth. Furthermore, noise was added to the prior distributions of the literature inferred parameters, by sampling them from a log uniform distribution spanning 1 order of magnitude (10×) around their literature inferred values (see Fig. S18 and S19 [https://github.com/segrelab/co_culture_device]). Even in the presence of this amount of noise in these values, the leakage stoichiometry parameter was effectively constrained by the data. Through this analysis, we integrated novel observations obtained with the BioMe plate with a computational model to infer plausible ranges of the leakage parameter governing the interaction between these auxotrophic E. coli mutants. This leakage parameter would be otherwise very difficult to measure experimentally. The posterior distributions obtained from this approximate Bayesian computation approach represent these inferences and their uncertainties, given the assumptions of our model and the data on which our estimates are based.

## DISCUSSION

We utilized the newly developed BioMe plate to observe a natural symbiotic interaction between organisms found in the D. melanogaster gut microbiome and to quantitatively study the interaction between two syntrophic engineered E. coli mutants. Our D. melanogaster results demonstrate the utility of BioMe to study a natural microbial interaction between two nonmodel organisms. This result largely corroborates recently published results (40, 51), although we identified commensal rather than mutualistic interactions between the studied *Lactobacillus* and *Acetobacter* species. This discrepancy is likely due to the use of a rich medium in our experiments which supplemented the amino acid auxotrophies in *Lactobacillus* and highlights the importance of context dependence in the characterization of interactions. The E. coli auxotroph interaction allowed us to integrate the data obtained with BioMe into a computational model providing quantitative insight into the parameters of this interaction. Moving forward, as computational modeling of microbial physiology advances, it may be possible to apply such modeling frameworks more ubiquitously. For example, genome-scale metabolic models could be incorporated into a spatial model of the BioMe device to represent the metabolic processes occurring in each interacting population and model emergent interactions (11). Such a model could be used, alongside BioMe observations, to gain quantitative, genome-scale insight into the interactions between microbial organisms, where the mechanism of interaction is not known *a priori*.

Our investigation of the E. coli lysine and isoleucine auxotrophs revealed that their syntrophic interaction was altered by the presence of a porous membrane separating the interacting auxotrophs. These results suggest that certain “high-stakes” interactions could be localized, such that highly proximal ecological neighbors reap the majority of the benefits from “leaked” communal resources. Leveraging a mechanistic, dynamical model for the syntrophic E. coli auxotroph coculture experiment, we inferred plausible leakage parameters that govern this localized interaction. This result corroborates and complements the recent imaging-based finding that pairs of E. coli proline and tryptophan auxotrophs can best help each other within a small local radius around each cell (53). While these local interactions may be dominated by diffusion-mediated processes, we cannot rule out, based on our experiment, that cross-feeding is also facilitated by contact-based mechanisms. Similar phenomena were reported, for example, in bacterium-fungus interactions, which were shown to require physical association for the activation of a cryptic biosynthetic pathway of secondary metabolism in the fungus (54). The local nature of these interactions may have important evolutionary and ecological consequences (55, 56), and the BioMe device is a promising platform for the continued study of such phenomena. For example, future work could focus on measuring the interactions between the remaining syntrophic auxotrophs from Mee et al. (46), beyond the lysine-isoleucine interaction investigated here.

There are some key limitations to the use of the BioMe plate. First, as is clear from our results, the porous membranes can have a significant impact on mutualistic cross-feeding interactions. In addition to altering the dynamics of metabolite exchange, the membrane limits interactions to only those mediated by diffusible molecules. Despite placing a constraint on the type of interactions that can be investigated, this limitation can be beneficial for the quantitative study and design of microbial consortia. For example, limiting interactions to those mediated by diffusible molecules makes it easier to robustly integrate and compare experimental measurements with metabolic models. Another limitation of the BioMe device is the fact that microbial crossover can occur across the membranes. In our syntrophic auxotrophs coculture experiment we observed microbial crossover in 2/9 pairwise assays with 0.03-, 0.1-, or 0.2-μm pore sizes, and 3/3 for the 0.4-μm pore size (see Fig. S7). These studies were exquisitely sensitive for detecting microbial crossover since the mutualistic interaction between the two strains would effectively amplify any crossed-over organisms. The observed crossovers may have arisen from E. coli crossing through the membrane pores, crossing around the membrane in an incompletely sealed device, or from standard cross-well contamination introduced by pipetting or other errors. The crossover observed in the 0.03- to 0.2-μm-pore-size membranes were more likely to have arisen from a faulty seal or contamination, rather than crossover through the pores, since these pore sizes are below the standard size used to filter sterilize bacterial growth medium and have been used successfully for other applications (35, 39). However, the consistent crossover in the 0.4-μm-pore-size membranes suggests that the bacteria were able to cross through the membrane. Although 0.4 μm is less than the typical diameter of an E. coli cell, E. coli is known to be able to pass through channels smaller than its own cell size through a growth/division driven processes (57). We opted to use the most conservative 0.03-μm-pore-size membranes for our D. melanogaster gut-associated bacteria experiments, and we suggest this pore size for other researchers using the device to observe interactions across a single membrane pore size. In addition, our modeling analysis of the E. coli auxotroph cocultures focused on the 0.1 μm pores size results, for which we did not measure any crossover. Future studies could focus on more thoroughly characterizing the crossover process for a variety of different organisms and conditions, and optimizing the design of the plate to limit contamination and crossover around the membrane. It should also be noted that the distribution of organisms in the device may not be homogeneous, leading to a potential bias in the optical density measurements. For example, in a mutualistic cross-feeding interaction the organisms may preferentially locate near the membrane or even attach to the membrane.

Regarding the manufacturing of the BioMe device, while the body segments can be fabricated using either milling or 3D printing methods, there is a significant trade-off between precision and flexibility in these two methods. We found that devices fabricated using milling had finer tolerances and were thus less prone to leakage than those that were 3D printed. However, the 3D printing approach facilitates rapid and easy prototyping of alternative plate layouts. Ultimately, users should choose the fabrication technique that best suits their goals.

Regarding the pore size dependence of small-molecule diffusion in the device, there was an interesting observation in the chemical dye diffusion experiments: the estimated diffusion rate was consistently higher when calculated using data from the input well where the dye was initially placed, as opposed to that of the opposite well. This effect was more pronounced for the larger pore sizes and for phenol red. A possible explanation for this effect is that the dye is settling or being sequestered in the membrane/device and thus appears to diffuse faster from the initial well and more slowly into the opposite well. This effect was not included in subsequent modeling efforts, since it produced only minor differences in the inferred diffusion rate relative to the range of uncertainty in the diffusion rate considered in our model. Furthermore, we note that the monotonic increase in diffusion rate may be related to the increase in open area percentage of the membranes (<1, 3, 10, and 19%, respectively, for the 0.03-, 0.1-, 0.2-, and 0.4-μm pore sizes, as reported on the Sterlitech “Performance by Pore Size” data sheet [https://www.sterlitech.com/hydrophilic-polycarbonate-membrane-filters.html]).

Although we demonstrate here the use of the BioMe plate to measure pairwise interactions, the device is a flexible technology that could be redesigned to incorporate multimember assays and higher-order interactions. 3D printing accessibility enables rapid fabrication and validation of potential plate layouts. Going beyond pairwise interactions, selected consortia of microbial organisms could be grown in each well of the BioMe plate to facilitate the investigation of higher-order interactions, which are thought to have an important impact on microbial community structure (58–60). Entire microbial communities could similarly be grown across from individual isolates to observe their growth supporting capabilities, as done with the iChip device (39), but with the added capability of observing growth dynamics. Furthermore, the 3D-printed design of the BioMe body segments could be modified to design novel interaction chambers, such as multiple wells connected to a central mixing chamber or sequential wells connected by porous membranes. Overall, the BioMe plate is relatively easy to manufacture and implement, and the use of this device to study the interaction of microbial organisms from a multitude of different contexts will help improve our understanding of and ability to engineer microbial communities.

## MATERIALS AND METHODS

### BioMe fabrication.

All materials required for BioMe fabrication are detailed in Table S1. O-rings, 6-32 flange nuts, 4-40 screws, and membranes were bought ready to use. Rods (6″ 6-32) were cut to 125 mm with a grind wheel. Stock food-grade silicone rubber sheets were laser-cut to gasket specifications using an Epilog Laser Mini 60W laser cutter; wrapping in dampened shop towels helped mitigate charring. The transparent base was CNC milled from clear polycarbonate sheets.

The body segments could either be machined or 3D printed. Two separate CNC milling operations were required to machine the vertical and horizontal features of the body segment from polypropylene sheet. A drill press was used to complete the spot holes, with each screw hole manually tapped with a 4-40 tap bit, and each body segment deburred and washed. Alternatively, stereolithography resin 3D-printing can be used to fabricate the body segments. FormLabs’ Form2 stereolithography printers were used with the Dental SG Resin, a biocompatible, autoclavable resin. Screw holes were manually tapped and body segments were sanded to size.

Files for the reproducible manufacturing of the BioMe device are available on GitHub (https://github.com/segrelab/co_culture_device in the “BioMe – Distribution Files” folder).

### Leakage test.

A simple visualization test was used to test water-tight seal throughout the assembled BioMe plate. All wells were loaded with 250 μL of 100 μM phenol red. The core BioMe device was then placed atop a paper towel and fit into the bottom tray and covered with the top lid. The BioMe plate was left in the shaking incubator overnight, and no leakage was verified.

### Sterilization protocol and validation.

To sterilize the BioMe plate after use, the device is disassembled, the membranes disposed of, and the components dishwashed and then autoclaved (gravity, 30-min exposure/15 min dry). For sterile assembly, a biosafety cabinet is recommended. Membranes are bathed in 70% ethanol for 30 min. Presterilized components are then sequentially assembled with the membranes under sterile conditions. For additional caution, presterilized device components may be ethanol bathed prior to assembly.

Sterilization protocol was validated to ensure no postcontamination. A sterilized reassembled BioMe plate was loaded with 250 μL of LB Miller media (10 g/L tryptone, 10 g/L NaCl, 5 g/L yeast extract) per well and placed into a 30°C static incubator for 72 h. A 10-μL aliquot from each well was then plated onto LB agar plates. The plates were incubated at 30°C for 72 h. No microbial growth was confirmed.

### Colorimetric dye diffusion measurement.

Calibration curves for phenol red (PR) and bromocresol purple (BP) were determined to relate the OD at the isosbestic point (478 nm for PR and 490 nm for BP) to concentration. A BioMe plate was assembled with membranes with no pores. Columns were loaded with 250 μL of various concentrations of dye: 450, 400, 350, 300, 250, 200, 150, 100, 50, and 0 μM; the top three rows were used for PR, and the bottom three rows for BP. The linear fit of the OD_isosbestic_ value versus the concentration data points was used for the calibration curve.

Diffusion experiments were used to estimate the diffusion rates for various membrane pore sizes for PR and BP. A BioMe plate was assembled with various membrane pore sizes at each body junction: no pores and 0.03-, 0.1-, 0.2-, and 0.4-μm pore sizes. Next, 250 μL of 400 μM dye was loaded into the left column, and 250 μL of distilled water was loaded into the right column for each pore size. The OD_isosbestic_ value was measured for 72 h at 15-min intervals.

Diffusion rates were estimated using a gradient-driven diffusion model (provided in Text S1). The time-dependent value on the left-hand side of the equation (see Text S1) was calculated from the data and fit to an exponential function (using MATLAB function fit with fittype: exp1) to infer our model’s diffusion rate for both dyes and pore sizes of 0.03, 0.1, 0.2, and 0.4 μm. The units for the diffusion rate, *d*, in our equations are L/h such that the flux has units of mmol/h. Our model is similar to Fick’s first law, in that there is a gradient driven diffusion; however, in our system the diffusion area and distance are fixed and thus lumped into the diffusion parameter giving rise to units that are different from a traditional “diffusion constant.”

### Amino acid diffusion measurement.

Diffusion rates of lysine and isoleucine through the porous membranes of the BioMe system were measured using colorimetric assay kits, a lysine assay kit (Cell BioLabs, Inc., MET-5130), and a branched-chain amino acid assay kit (Cell BioLabs, Inc., MET-5056). A BioMe device was assembled with 0.1-μm-pore-size membranes, and stock solutions of 400 μM lysine and isoleucine in phosphate-buffered saline (PBS) were prepared. The left wells of the BioMe were loaded with 250 μL of the respective amino acid samples, and the right wells were loaded with 250 μL of PBS. At 1.5, 3, 6, 12, and 24 h, the entire volumes of both left and right wells (*n* = 3) were sampled and stored at −80°C; aliquots from the original amino acid stock solutions and PBS were also stored. Once all samples were collected, a linear-fit standard curve was measured and calculated for lysine (100, 50, 25, 12.5, 6.25, and 0 μM in duplicates) and isoleucine (1,000, 500, 250, 125, 62.5, 31.25, 15.63, and 0 μM in triplicates) at 550 nm and 450 nm OD, respectively (see Fig. S3A). The amino acid concentrations in the experimental samples were measured using their respective colorimetric assay, according to the manufacturer’s protocol. Due to the recommended linear range of the lysine assay kit (0 to 100 μM), all experimental samples for lysine were diluted 4× in PBS. The ΔOD values between experimental and negative controls were compared to the standard curve to extrapolate the amino acid concentration. The time course of amino acids diffusing from the left to right well were then calculated and plotted (see Fig. S3B). The diffusion rate was estimated, as was done for the dye diffusion rates, by fitting an exponential function (see Text S1) to all of the points from either the left or right wells.

### *Drosophila* gut microbiome interaction.

Strains of Acetobacter oryzifermentans, Lactobacillus brevis, and Lactobacillus plantarum were isolated as previously described (61) and streaked onto YPD agar plates (10 g/L peptone, 10 g/L yeast extract, 8 g/L dextrose, 15 g/L agar). The identity and lack of cross-contamination were confirmed by colony PCR using species-specific primers and gel electrophoresis (45). For each strain, four clonal replicates were picked from colonies, and grown in 5 mL of YPD broth (10 g/L peptone, 10 g/L yeast extract, 8 g/L dextrose) at 30°C in a static incubator for 20 h. Cells were centrifuged, and pellets were washed three times in PBS. The OD_600_ values for 250 μL of each culture (C) and PBS blanks (PBS) were read. Cultures were diluted with YPD media to an OD_600_ of 0.1 using the following formula:

$$\%\text{culture in dilution }=\frac{{\text{set OD}}_{\text{600}}\;-\;{\text{PBS OD}}_{\text{600}}}{{\text{culture OD}}_{\text{600}}\;-\;{\text{PBS OD}}_{\text{600}}}=\frac{0.1\;-\;\text{PBS}}{C\;-\;\text{PBS}}$$

Dilutions were redone for those not within 10% deviation. Cultures were further diluted 1:100 in YPD media. Assembled BioMe plate, with 0.03-μm-pore-size membranes, was loaded with diluted cultures in appropriate wells and sealed with Parafilm. The plate was then run on a plate reader for 96 h at 30°C, with OD_600_ measurements obtained at 15-min intervals with no shaking.

### Syntrophic coculture interaction.

Diffusion of amino acids through membranes with various pore sizes was validated using Lambda Red-recombineered EcNR1 E. coli for knockout of LysA (ΔLys) and IlvA (ΔIle) (46). Strains were streaked and selected on LB+Cam (25 μg/mL chloramphenicol) agar plates. Strains were grown in 5 mL of LB+Cam broth (*n* = 4 for both ΔLys and ΔIle) in a 12-well plate for 24 h inside the plate reader, with OD_600_ measurements obtained every 15 min. These growth curves were used to determine the dilution time (*t*_dil_ = 6 h) for proceeding experiments (see Fig. S15 [https://github.com/segrelab/co_culture_device]).

For amino acid diffusion experiment, a BioMe plate was assembled with various membrane pore sizes at each body junction: no pores and 0.03-, 0.1-, 0.2-, and 0.4-μm pores. For either strain, six clonal replicates were grown in 5 mL of LB+Cam broth at 30°C in a static incubator for 6 h and then washed in minimal medium (M9 + glucose [0.4%] + thiamine [1 μg/mL] + biotin [1 μg/mL] + chloramphenicol [25 μg/mL]). Cultures were diluted with minimal media to an OD_600_ of 0.1 using the formula provided for the *Drosophila* gut microbiome interaction methods. Cultures were further diluted 1:100 in both minimal media and minimal media supplemented with lysine (0.0134%) or isoleucine (0.0065%) for ΔLys or ΔIle, respectively. For all experiments using minimal media supplemented with amino acids (aa), the amino acid concentration was calculated such that a theoretical yield of 10^9^ cells would be reached: for lysine, (1.1 × 10^8^ aa/cell) × (10^9^ cells)/[(6.022 × 10^23^ aa/mol) × (250 × 10^−6^ L)] = 7.3065 × 10^−4^ mol/L; and for isoleucine, (7.5 × 10^7^ aa/cell) × (10^9^ cells)/[(6.022 × 10^23^ aa/mol) × (250 × 10^−6^ L)] = 4.98 × 10^−4^ mol/L. The values for amino acids per cell (aa/cell) were obtained from Mee et al. (46). A sterile assembled BioMe plate was loaded with diluted cultures in appropriate wells; for each pore size, *n* = 4 for the amino acid diffusion assay, *n* = 1 for the negative control with no supplemental amino acid, and *n* = 1 for the positive control with an auxotroph loaded with supplemental amino acid in same well. The BioMe plate was run on the plate reader for 96 h at 30°C, with OD_600_ measurements obtained at 15-min intervals with no shaking.

Similar growth, wash, and dilution procedures were followed for the syntrophic coculture experiment. For each pore size, *n* = 3 for the coculture assay across the membrane, *n* = 1 for both negative controls without a coculture partner, and *n* = 1 for the positive control with coculture partners in the same well. The plate reader was run with a BioMe plate for 96 h at 30°C, with OD_600_ measurements at 15-min intervals. At the end of the kinetic read, a 5-μL aliquot from each well was cultured on minimal medium agar plates supplemented with either lysine or isoleucine to determine instances of crossover. Plates were cultured for 72 h at 30°C in a static incubator.

### Computational modeling overview.

We implemented a mathematical model that uses ordinary differential equations (ODE) to describe the dynamic changes in the abundance of the two interacting organisms and the key metabolites necessary for their growth (see Text S1). Each compartment in the BioMe can harbor different amounts of organisms and metabolites, encoded by distinct variables in our differential equations model. Changes in the population of each organism in each compartment can only occur due to growth. This is described by a Monod model in which the rate is determined by the limiting nutrient, which can be either glucose or an essential amino acid leaked by the other organism. Metabolite concentrations in each compartment, on the other hand, can change due to consumption or secretion by a given organism or due to diffusion across the porous membrane. The equations describing the system are based on standard mass action kinetics. For diffusion of metabolites across the membrane, we assume (based on Fick’s first law) that the flux is proportional to the concentration gradient across the two compartments, with a diffusion rate that we inferred experimentally. The parameters for the uptake of metabolites and stoichiometry (biomass produced per g of glucose and g of amino acid) were inferred from the literature. The systems of differential equations can be solved numerically for any given set of kinetic parameters and initial conditions. One important parameter that could not be easily measured or inferred from the literature is the rate of amino acid leakage from each bacterium. In order to infer these parameters, we implemented a probabilistic computational approach (approximate Bayesian computation) on top of the ODE model, which identifies the leakage parameter range that is consistent with our experimental data.

### Computational modeling details.

We developed a computational model to simulate the interaction between E. coli amino acid auxotrophs in the BioMe device. The model consists of two wells, each with a given volume. Each well can start with any defined amount of cells (isoleucine and lysine auxotrophs), initial amino acids (isoleucine and lysine), and glucose. The glucose and growth-supporting amino acids are taken up by the cells as they grow, the non-growth-supporting amino acids are secreted, and all metabolites can diffuse between the two wells. Metabolite diffusion is modeled by a gradient driven flux across the porous membrane. Metabolite uptake flux is bounded by Michaelis-Menten kinetic uptake equations, and growth rate is determined by the minimum biomass flux that can be produced based on metabolite uptake rates and biomass stoichiometry. Amino acid leakage is determined by a stoichiometric parameter specifying the amount of amino acid leaked into the environment for each gram of E. coli biomass produced. The uptake, growth constraints, and leakage components of this model are analogous to previously developed dynamic flux balance analysis models (11). Thus, our model is essentially a simplified dynamic flux balance analysis model with a growth rate proportional leakage term and gradient driven diffusion between compartments. The full model dynamics are presented in Text S1, and the code is available online (https://github.com/segrelab/co_culture_device). Simulation of the model was implemented in MATLAB. Testing of runtime and accuracy was conducted to benchmark three MATLAB ODE solvers (ode23tb, ode45, and ode15s). The function ode23tb was found to have the best performance, with consistent accuracy when using maximum step sizes of 0.0025 h. Thus, this solver function and maximum step size setting was used to simulate model dynamics.

We implemented an approximate Bayesian computation approach to integrate experimental data with computational modeling. Approximate Bayesian computation is used to infer the posterior distribution of the parameters of a computational model by using simulations of experimental results to approximate the likelihood of the data (62, 63). We implemented a rejection-based algorithm where sample parameters are initially drawn from a given prior distribution and are included in the posterior distribution if the difference between the simulated and experimental value of a given statistic is less than some specified threshold. The statistic that we utilized was the log base 10 of the ratio between growth at 48 h of the E. coli auxotrophs in the same well (positive control) divided by the growth in opposite wells (membrane separated) (see Text S1). Using this ratio allowed us to compare experimental results to simulated results without calibrating between biomass units, since our OD growth measurements were made ratio scale by subtracting a blank control. The threshold that we used was 2× the standard deviation from the experimentally measured mean ratio for the equal leakage model, and 10× the standard deviation for the unequal leakage model.

We began by sampling diffusion and leakage parameters while assuming that the leakage of isoleucine and lysine were equal (Fig. 7). The uptake kinetics and biomass parameters were estimated from the literature and were fixed at their estimated values (prior distribution was a Dirac delta function) (see Text S1). The leakage and diffusion parameters were randomly sampled from a log uniform prior distribution: the diffusion rate ranging 1 order of magnitude around the mean experimentally measured value for lysine and isoleucine (10^−4.911^ to 10^−3.911^ L/hr), and the leakage stoichiometry ranging from 10^−2^ to 10 mmol/g. Next, we used our model to investigate the unequal growth of the ΔLys and ΔIle auxotrophs (see Fig. S8). We fixed the diffusion rate to the mean measured value for lysine and isoleucine (3.88*10^−5^ L/h). Then, we sampled the leakage for ΔLys and ΔIle independently from log uniform prior distributions with the same ranges as used previously. Finally, we added noise to all the literature-estimated parameters (with the exception of volume) to represent an increased level of uncertainty (see Fig. S18 and S19 [https://github.com/segrelab/co_culture_device]). We sampled all literature-estimated parameters from a log uniform prior distribution that varied by 1 order of magnitude around the originally fixed literature estimated value. This more uncertain prior distribution was used to repeat the inference of leakage stoichiometry for both equal (see Fig. S18 [https://github.com/segrelab/co_culture_device]) and unequal (see Fig. S19 [https://github.com/segrelab/co_culture_device]) cases.

### Data availability.

All data and codes are available on GitHub at https://github.com/segrelab/co_culture_device. The data includes raw kinetic OD measurements for the small molecule dye diffusion measurement, amino acid diffusion measurement, *Drosophila* gut microbe coculture, amino acid diffusion, and syntrophic E. coli auxotroph coculture experiments, as well as the MATLAB scripts for the generation of all figures from raw data are provided in the “raw_data_and_plots” subdirectory. MATLAB scripts for solving the differential equations of the computational model and for the Bayesian analyses are located in the “modeling” subdirectory. Files for the reproducible manufacturing of the BioMe device are available on GitHub in the “BioMe – Distribution Files” folder.

10.1128/mSystems.00017-21.9 FIG S8 Sampling of unequal leakage parameters. (A) Sample space of ΔLys and ΔIle leakage. The diffusion rate was set to the experimentally measured mean diffusion rate for lysine and isoleucine 3.88 × 10^−5^ L/h. Here, the log base 10 of the ratio between positive-control growth and membrane-separated growth (color bar) for ΔLys is shown. The ΔIle ratio plot looks similar with the *x* and *y* axis transposed. Sampled points within 10× the experimentally measured ratio standard deviation from the mean for both ΔLys and ΔIle (ΔLys: standard deviation, 0.015; mean, 0.925; ΔIle: standard deviation, 0.026; mean, 1.35) were used to approximate the posterior distribution of the leakage stoichiometries through approximate Bayesian computation (histograms, black, prior distribution; gold, posterior distribution). (B) Example growth curves for different regions of the parameter space. Membrane separated lysine (blue) and isoleucine (green) auxotrophs, and same-well positive control (gray) growth curves are shown. Download FIG S8, JPG file, 1.5 MB. Copyright © 2023 Jo et al. 2023 Jo et al. https://creativecommons.org/licenses/by/4.0/ This content is distributed under the terms of the Creative Commons Attribution 4.0 International license.
