# Supplementary figures and images for: Construction and Modeling of a Coculture Microplate for Real-Time Measurement of Microbial Interactions
Source: mSystems. 2023 Feb 21;8(2):e00017-21. doi: 10.1128/msystems.00017-21 (PMC10134821; doi:10.1128/msystems.00017-21)

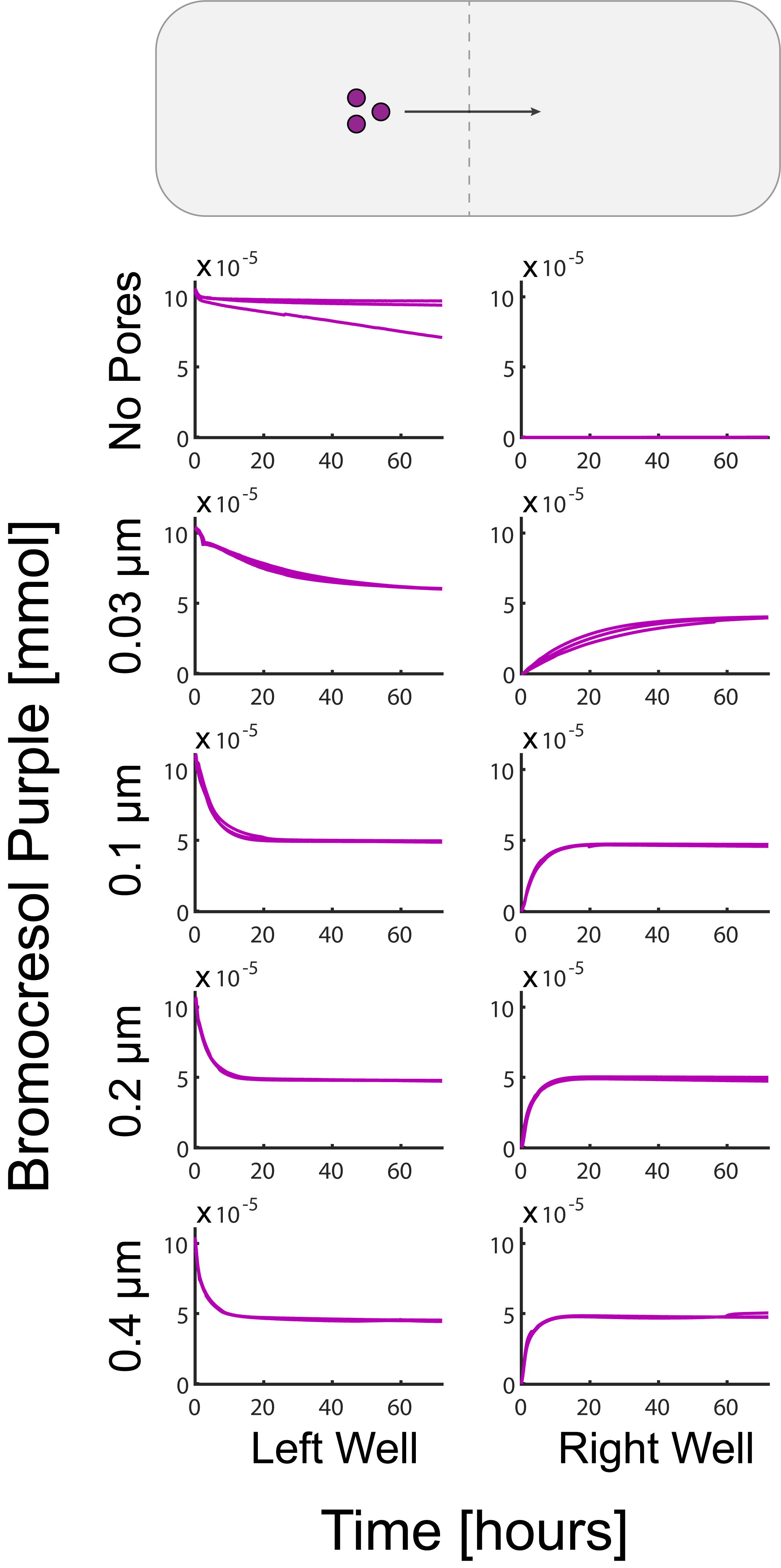

Supplement: FIG S1 [file msystems.00017-21-s0003.jpg]

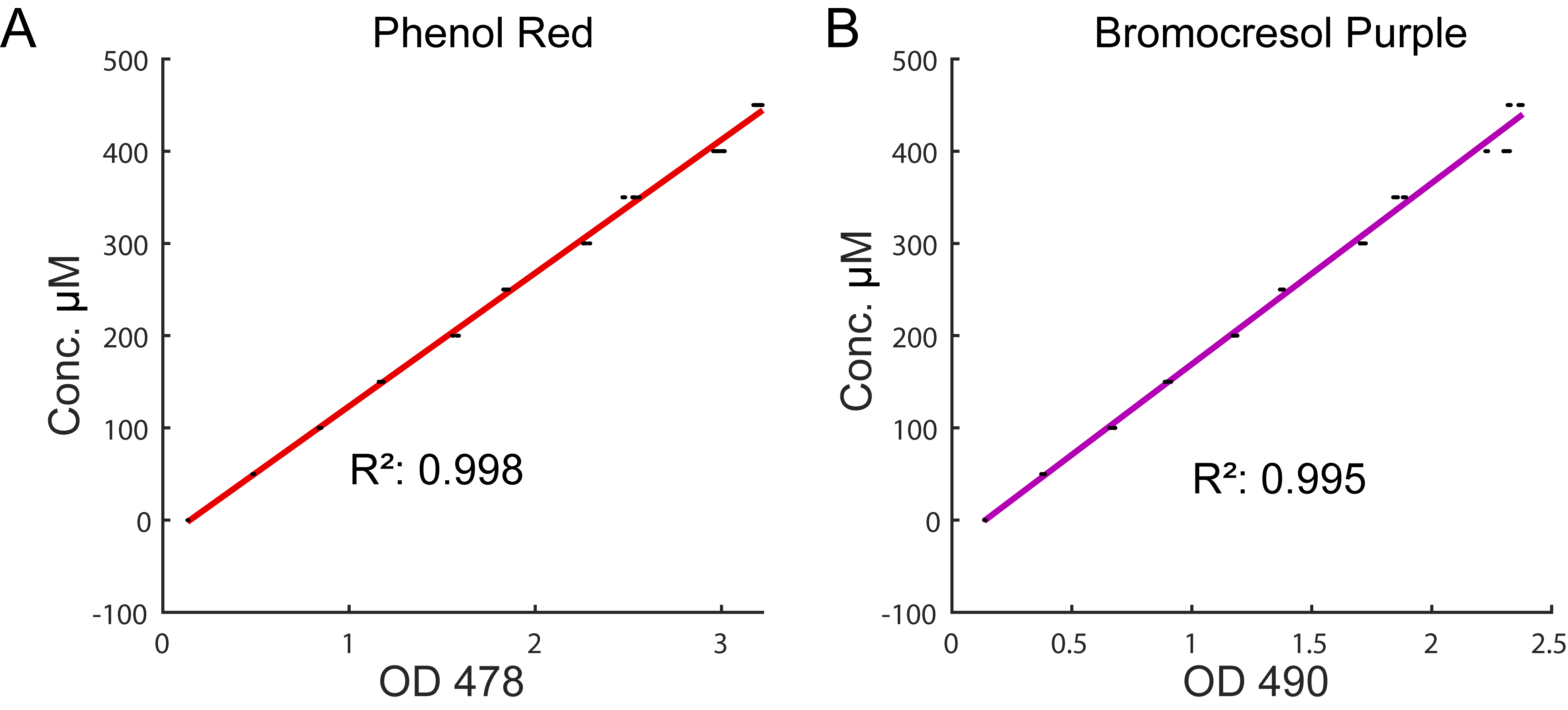

Supplement: FIG S2 [file msystems.00017-21-s0004.jpg]

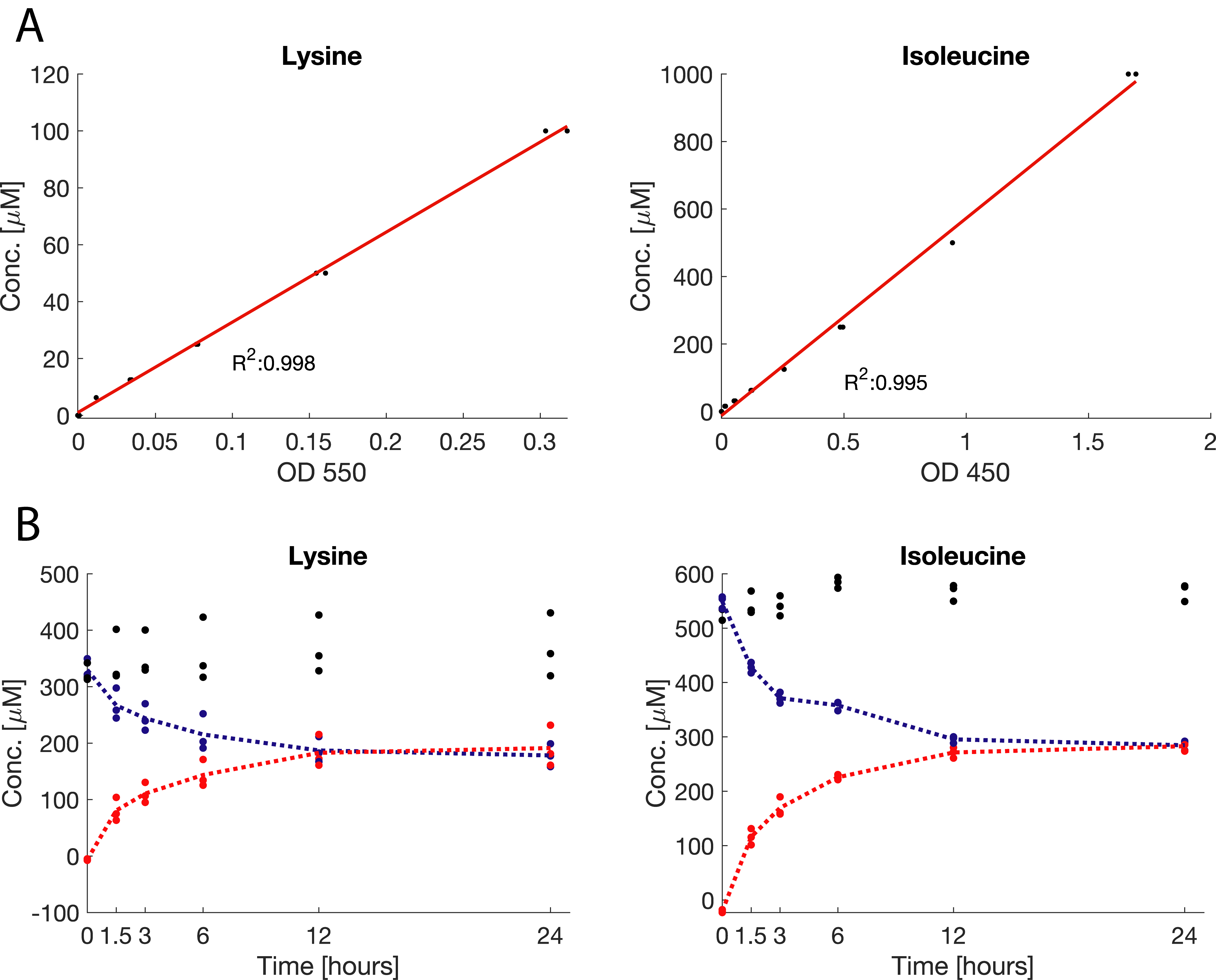

Supplement: FIG S3 [file msystems.00017-21-s0005.jpg]

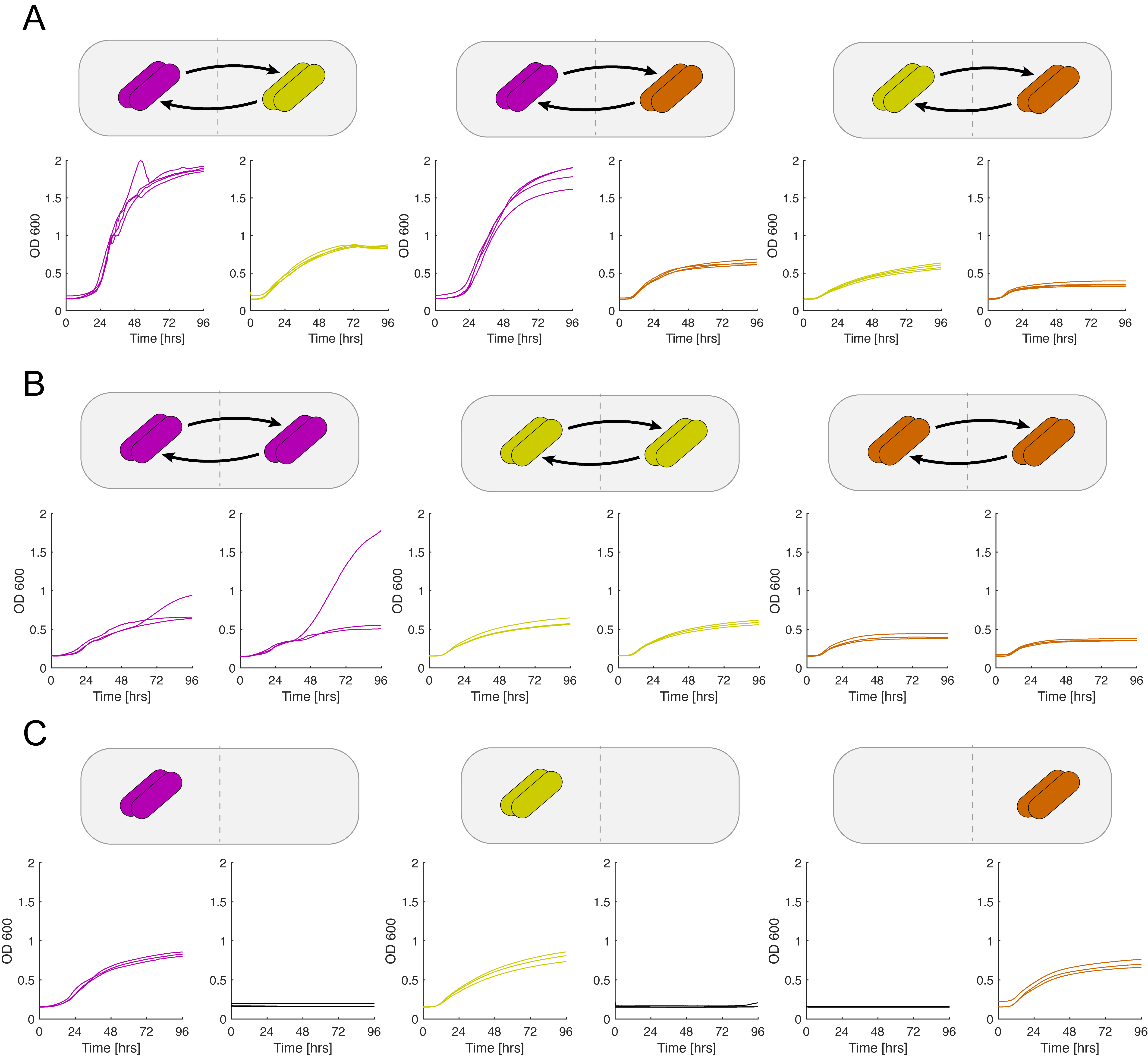

Supplement: FIG S4 [file msystems.00017-21-s0006.jpg]

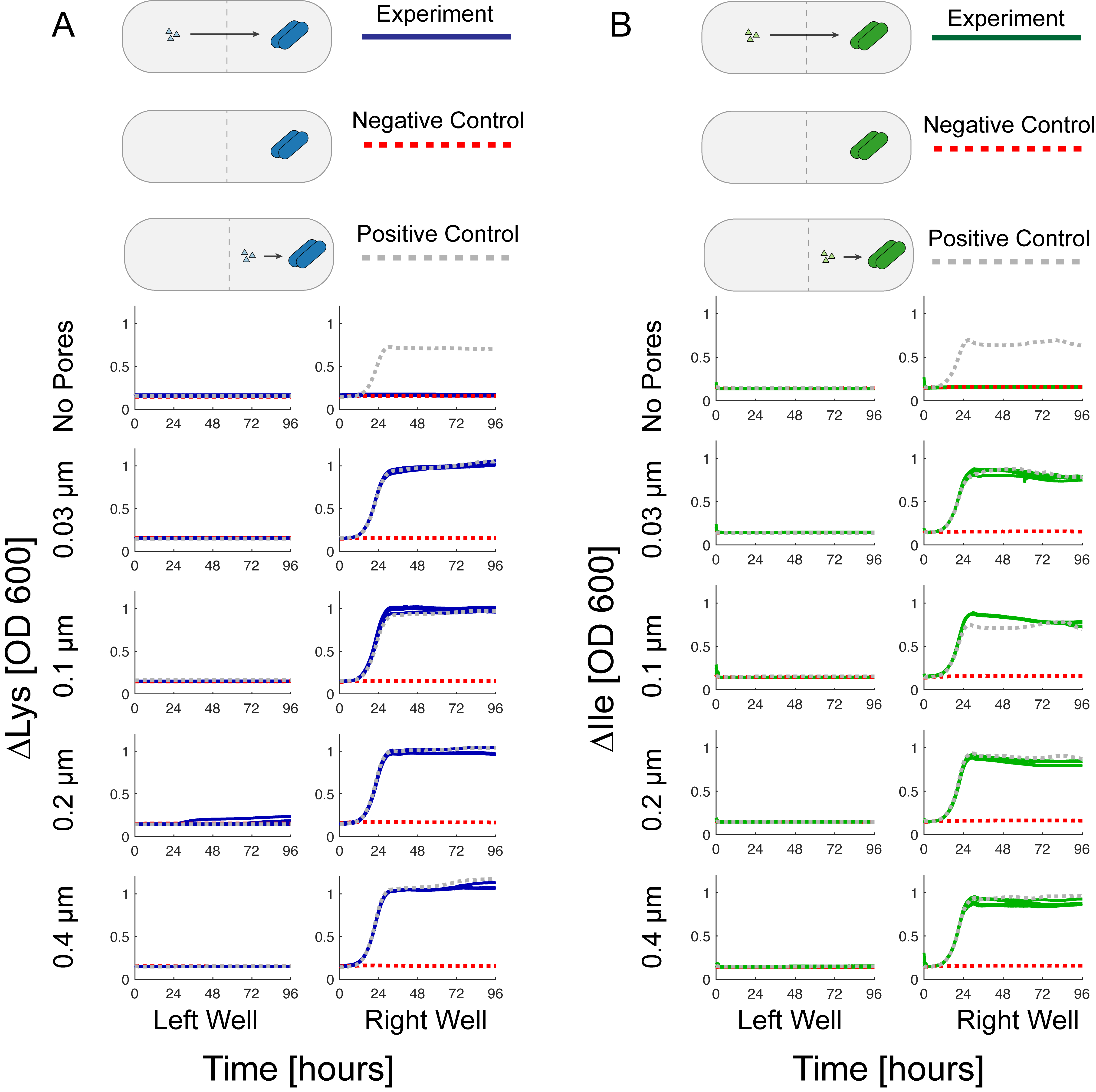

Supplement: FIG S5 [file msystems.00017-21-s0007.jpg]

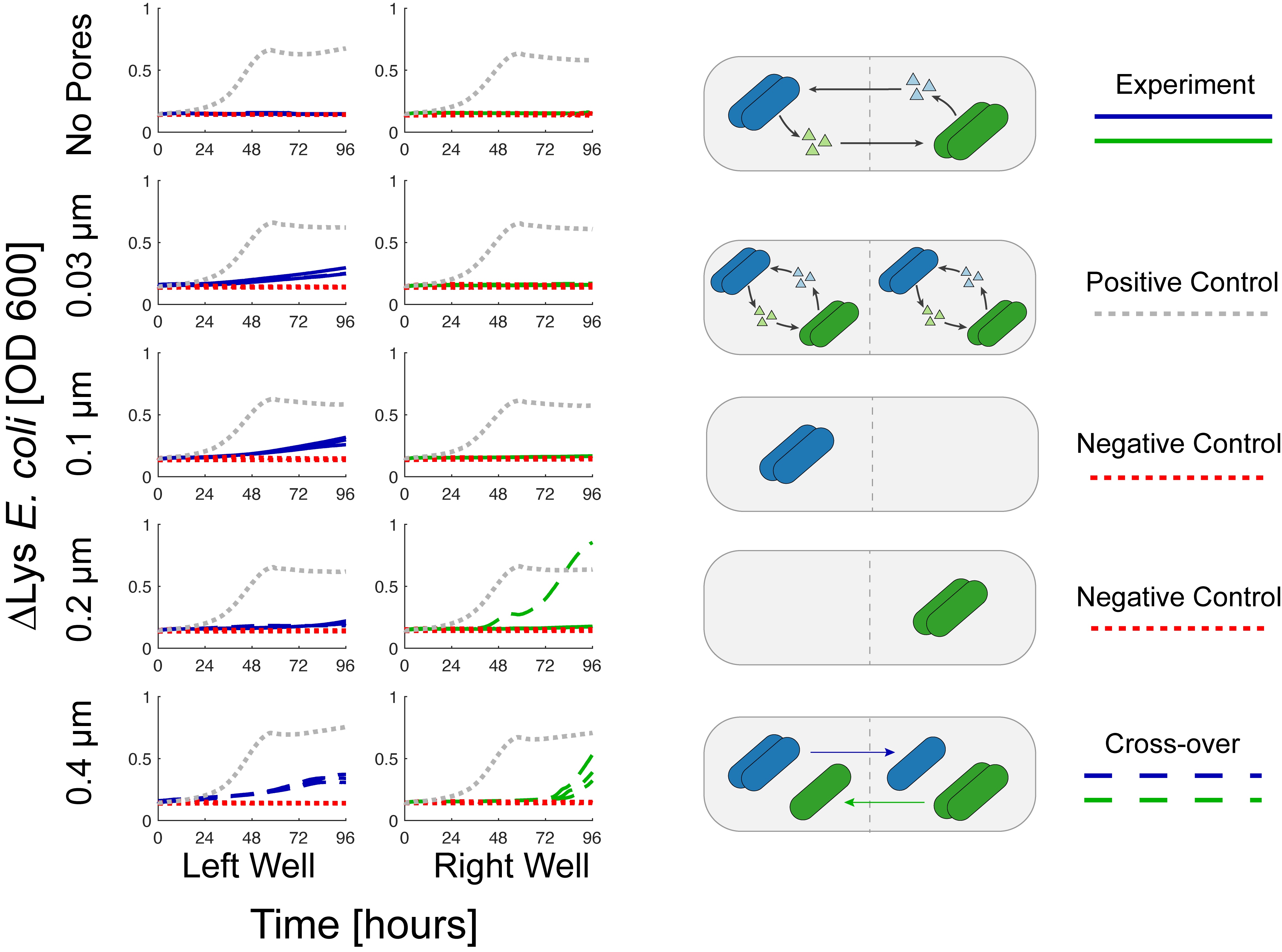

Supplement: FIG S6 [file msystems.00017-21-s0008.jpg]

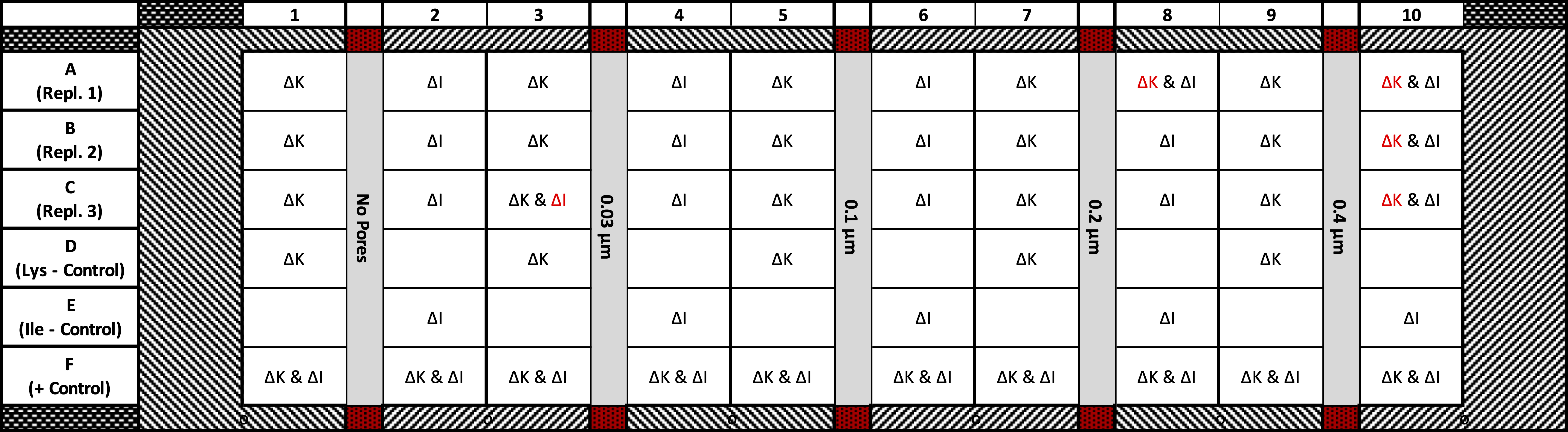

Supplement: FIG S7 [file msystems.00017-21-s0009.jpg]

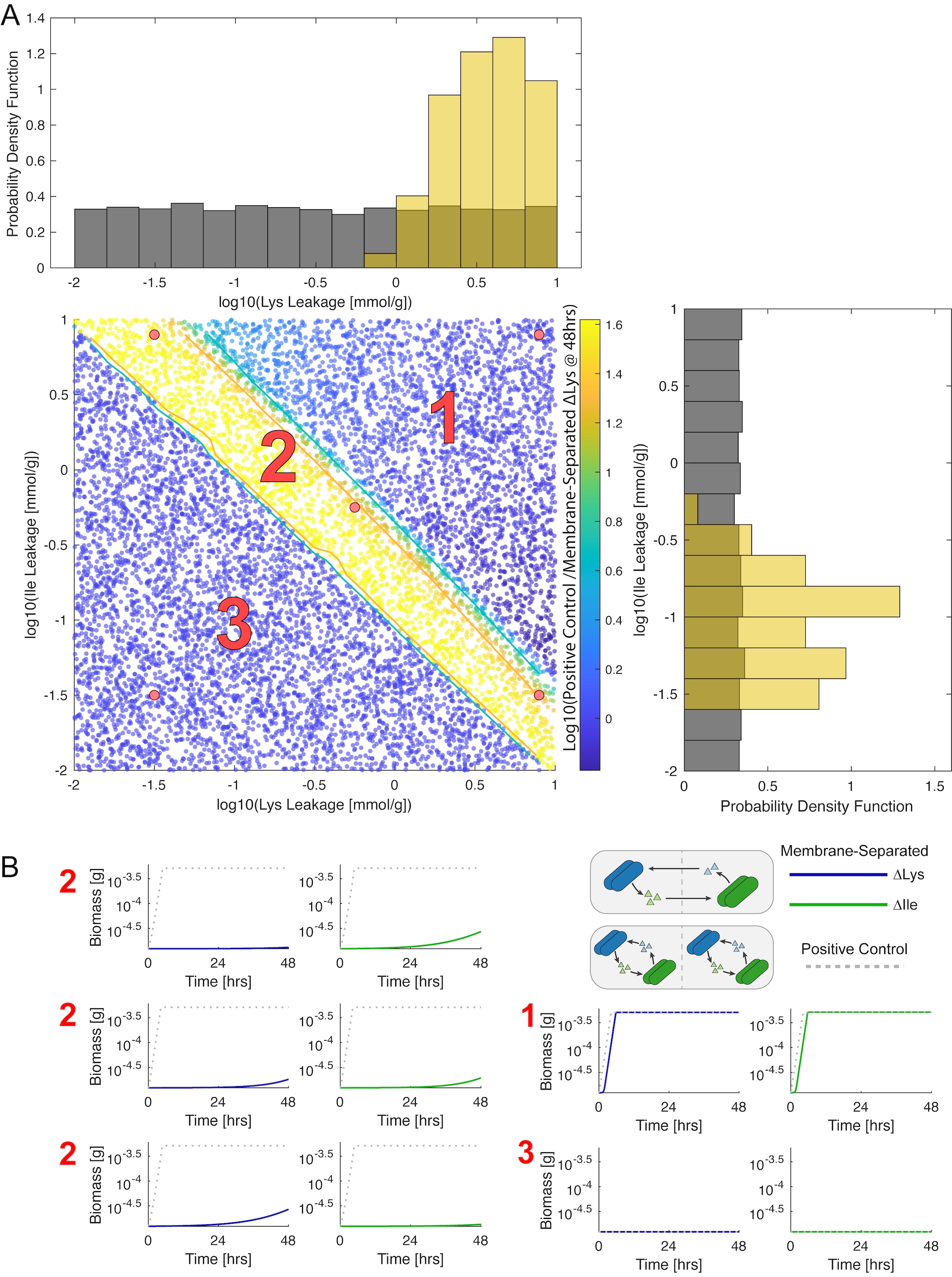

Supplement: FIG S8 [file msystems.00017-21-s0010.jpg]
