# Supplementary material for: Construction and Modeling of a Coculture Microplate for Real-Time Measurement of Microbial Interactions
Source: mSystems. 2023 Feb 21;8(2):e00017-21. doi: 10.1128/msystems.00017-21 (PMC10134821; doi:10.1128/msystems.00017-21)
Supplement: TEXT S1 [file msystems.00017-21-s0001.docx]

**Supplemental Text S1: Supplemental Equations**

**(A) Metabolite diffusion dynamics**

We derived an equation for the diffusion of molecules across the membrane by simplifying our overall model to include only the diffusion component and integrating. We show the derivation of the amount of amino acid in the left well (the well with the initial placement of amino acid) below. The derivation for the right well is the same with the rate of change of amino acid set to a positive and the initial concentration set to 0.

$$V_{D}^{A}=d*\left( \frac{A_{L}}{v}-\frac{A_{R}}{v} \right)$$

$$\frac{dA_{L\{R\}}}{dt}=-\{+\}V_{D}^{A}$$

$$\frac{dA_{L}}{dt}=-V_{D}^{A}=-d*\left( \frac{A_{L}-A_{R}}{v} \right)$$

$$A_{R}=K-A_{L}$$

$$\frac{dA_{L}}{dt}=-V_{D}^{A}=-d*\left( \frac{A_{L}-(K-A_{L})}{v} \right)$$

$$\frac{dA_{L}}{dt}=-d*\left( \frac{2A_{L}-K}{v} \right)$$

$$\frac{1}{2A_{L}-K}dA_{L}=\frac{-d}{v}dt$$

$$\frac{1}{2}ln\left( 2A_{L}(t)-K \right)+c1=\frac{-d}{v}t+c2$$

$$2A_{L}(t)-K={Ce}^{-2\frac{d}{v}t}$$

$$A_{L}\left( 0 \right)=K$$

$$2K-K={Ce}^{-2\frac{d}{v}0}$$

$$C=K$$

$$2A_{L}(t)-K={Ke}^{-2\frac{d}{v}t}$$

$$A_{L}(t)-\frac{K}{2}={\frac{K}{2}e}^{-2\frac{d}{v}t}$$

$$\boldsymbol{A}_{\boldsymbol{L\{R\}}}\left( \boldsymbol{t} \right)\boldsymbol{-}\frac{\boldsymbol{K}}{\boldsymbol{2}}\boldsymbol{=\{-\}}\frac{\boldsymbol{K}}{\boldsymbol{2}}\boldsymbol{e}^{\boldsymbol{-2}\frac{\boldsymbol{d}}{\boldsymbol{v}}\boldsymbol{t}}$$

$$A_{L\{R\}}\left( t \right):the amount of metabolite in the left \left\{ right \right\} well at time t [mmol]$$

$$K:the initial total amount of metabolite [mmol]$$

$$d:diffusion rate [L/hr]$$

$$v:volume of a single well (left and right wells are assumed to have the same volume) [L]$$

**(B) Computational Model of E. coli Amino Acid Auxotroph Interaction**

Variables [units] (notes):
$G_{L\{R\}}$ [mmol] (glucose left{right})
$K_{L\{R\}}$ [mmol] (Lysine left{right})
$I_{L\{R\}}$ [mmol] (Isoleucine left{right})
$B_{L\{R\}}^{K}$ [g] (Biomass of *E. coli* ΔLysine left{right})
$B_{L\{R\}}^{I}$ [g] (Biomass of *E. coli* ΔIsoleucine left{right})

Parameters [units]:
Kinetics
${vmax}_{G}$ [mmol/(hr*g)]
${vmax}_{K}$ [mmol/(hr*g)]
${vmax}_{I}$ [mmol/(hr*g)]

${km}_{G}$ [mmol/L]

${km}_{K}$ [mmol/L]

${km}_{I}$ [mmol/L]

Biomass Stoichiometry

$z_{G}$ [g/mmol]

$z_{K}$ [g/mmol]

$z_{I}$ [g/mmol]

Leakage Stoichiometry

$y_{K}$ [mmol/g]

$y_{I}$ [mmol/g]

Volume

$v$ [L]

Diffusion Rate

$d$ [L/hr]

Fluxes:
Growth

$$V^{B_{L\left\{ R \right\}}^{K}}=\min\left( \frac{{vmax}_{G}*\frac{G_{L\left\{ R \right\}}}{v}}{{km}_{G}+\frac{G_{L\left\{ R \right\}}}{v}}*z_{G},\frac{{vmax}_{K}*\frac{K_{L\left\{ R \right\}}}{v}}{{km}_{K}+\frac{K_{L\left\{ R \right\}}}{v}}*z_{K} \right)$$

$$V^{B_{L\left\{ R \right\}}^{I}}=\min\left( \frac{{vmax}_{G}*\frac{G_{L\left\{ R \right\}}}{v}}{{km}_{G}+\frac{G_{L\left\{ R \right\}}}{v}}*z_{G},\frac{{vmax}_{I}*\frac{I_{L\left\{ R \right\}}}{v}}{{km}_{I}+\frac{I_{L\left\{ R \right\}}}{v}}*z_{I} \right)$$

Consumption
$V_{B_{L\{R\}}^{K}}^{G}=\frac{V^{B_{L\left\{ R \right\}}^{K}}}{z_{G}}$

$$V_{B_{L\{R\}}^{I}}^{G}=\frac{V^{B_{L\left\{ R \right\}}^{I}}}{z_{G}}$$

$$V_{B_{L\{R\}}^{K}}^{K}=\frac{V^{B_{L\left\{ R \right\}}^{K}}}{z_{K}}$$

$$V_{B_{L\{R\}}^{I}}^{I}=\frac{V^{B_{L\left\{ R \right\}}^{I}}}{z_{I}}$$

Leakage

$$V_{B_{L\{R\}}^{I}}^{K}=V^{B_{L\left\{ R \right\}}^{I}}*y_{K}$$

$$V_{B_{L\{R\}}^{K}}^{I}=V^{B_{L\left\{ R \right\}}^{K}}*y_{I}$$

Diffusion

$$V_{D}^{G}=d*\left( \frac{G_{L}}{v}-\frac{G_{R}}{v} \right)$$

$$V_{D}^{K}=d*\left( \frac{K_{L}}{v}-\frac{K_{R}}{v} \right)$$

$$V_{D}^{I}=d*\left( \frac{I_{L}}{v}-\frac{I_{R}}{v} \right)$$

Dynamics:

$$\frac{dG_{L\{R\}}}{dt}=-V_{B_{L\left\{ R \right\}}^{K}}^{G}*B_{L\left\{ R \right\}}^{K}-V_{B_{L\left\{ R \right\}}^{I}}^{G}*B_{L\left\{ R \right\}}^{I}-\{+\}V_{D}^{G}$$

$$\frac{dK_{L\{R\}}}{dt}=-V_{B_{L\left\{ R \right\}}^{K}}^{K}*B_{L\left\{ R \right\}}^{K}+V_{B_{L\left\{ R \right\}}^{I}}^{K}*B_{L\left\{ R \right\}}^{I}-\{+\}V_{D}^{K}$$

$$\frac{dI_{L\{R\}}}{dt}=-V_{B_{L\left\{ R \right\}}^{I}}^{I}*B_{L\left\{ R \right\}}^{I}+V_{B_{L\left\{ R \right\}}^{K}}^{I}*B_{L\left\{ R \right\}}^{K}-\{+\}V_{D}^{I}$$

$$\frac{dB_{L\{R\}}^{K}}{dt}=V^{B_{L\left\{ R \right\}}^{K}}*B_{L\{R\}}^{K}$$

$$\frac{dB_{L\{R\}}^{I}}{dt}=V^{B_{L\left\{ R \right\}}^{I}}*B_{L\{R\}}^{I}$$

**(C) Parameters and Initial Conditions**

Literature derived parameters:

Kinetics
These parameters were estimated from the literature as in Harcombe *et al.* cell reports 2014 (1); the original source referenced in this paper was Gosset, microbial cell factories 2005 (2).

- ${vmax}_{G}=10$ [(mmol G)/(hr*(grams *E. coli*))]
- ${vmax}_{K}=10$ [(mmol K)/(hr*(grams *E. coli*))]
- ${vmax}_{I}=10$ [(mmol I)/(hr*(grams *E. coli*))]
- ${km}_{G}=0.01$ [(mmol G)/Liter]
- ${km}_{K}=0.01$ [(mmol K)/Liter]
- ${km}_{I}=0.01$ [(mmol I)/Liter]

Biomass Stoichiometry

Yeild of *E. coli* on glucose: 0.5 [grams *E. coli* / gram glucose] (from BioNumbers 105318, Shiloach et al. Biotechnol Adv. 2005)(3)

Molar mas of glucose: 180.156e-3 [grams glucose / mmol glucose]

- $z_{G}=0.0901$ [grams *E. coli* / mmol glucose]

Yeild of *E. coli* on amino acids (from Mee et al. PNAS. 2014 supplementary dataset S01) (4):

1.1e8 [# K / cell *E. coli*]

7.5e7 [# I / cell *E. coli*]

Avogadro’s number:
6.022e20 [# / mmol]

Gram / cell *E. coli* (from cell biology by the numbers “how big is an *E. coli* cell and what is its mass” cell mass varies with growth rate and the number chosen is in the middle of the *E. coli* growth rate range) (5):

500e-15 [gram *E. coli* / cell *E. coli*]

- $z_{K}=2.7373$ [g *E. coli* /mmol K]
- $z_{I}=4.0148$ [g *E. coli* /mmol I]

Initial Conditions:

Glucose

Stock solution

20 [g glucose] / 100 [mL stock]

Concentration

2 [mL stock] / 100 [mL medium]

Molecular weight

180.156e-3 [g/mmol]

Volume

250e-3 [mL medium]

- $G=5.55*{10}^{-3}$ [mmol] (glucose)

Lysine

Yeild of *E. coli* on amino acids (from Mee et al. PNAS. 2014 supplementary dataset S01) (4):

1.1e8 [# K / cell *E. coli*]

Targeted cell count of 10^9^

Avogadro’s number:
6.022e20 [# / mmol]

- $K=1.83*{10}^{-4}$ [mmol] (Lysine)

Isoleucine

Yeild of *E. coli* on amino acids (from Mee et al. PNAS. 2014 supplementary dataset S01) (4):

7.5e7 [# I / cell *E. coli*]

Targeted cell count of 10^9^

Avogadro’s number:
6.022e20 [# / mmol]

- $I=1.25*{10}^{-4}$ [mmol] (Isoleucine)

Biomass ΔLysine and ΔIsoleucine

Average final OD from positive controls: 0.635 [OD]

Theoretical yield from glucose stoichiometry: 5.00 * 10^-4^ [g *E. coli*]

Average initial OD from negative controls: 0.151 [OD]

Average blank OD from negative controls: 0.139 [OD]

Initial biomass:

$$\frac{5.001*{10}^{-4}\left[ g E. coli \right]}{0.6349-0.1389 \left[ OD \right]}*\left( 0.1511-0.1389 \left[ OD \right] \right)$$

- $B=1.23*{10}^{-5}$ [g *E. coli*]

Stock culture OD600: 0.1

Blank media OD600: 0.04

Inoculation dilution: 1/100

Rough calibration of *E. coli* OD to cells/mL: 8*10^8^ cell/mL / 1 OD 600

Gram / cell *E. coli* (from cell biology by the numbers “how big is an *E. coli* cell and what is its mass” cell mass varies with growth rate and the number chosen is in the middle of the *E. coli* growth rate range):

500e-15 [gram *E. coli* / cell *E. coli*]

Volume: 250*10^-3^ mL

- $B^{K}=6*{10}^{-8}$ [g] (Biomass of *E. coli* ΔLysine)

Biomass ΔIsoleucine

Same inoculation amount as ΔLysine

- $B^{I}=6*{10}^{-8}$ [g] (Biomass of *E. coli* ΔIsoleucine)

Experimentally derived parameters:

Volume

$v={250*10}^{-6}$ [L]

1. Harcombe WR, Riehl WJ, Dukovski I, Granger BR, Betts A, Lang AH, Bonilla G, Kar A, Leiby N, Mehta P, Marx CJ, Segrè D. 2014. Metabolic resource allocation in individual microbes determines ecosystem interactions and spatial dynamics. Cell Rep 7:1104–1115.

2. Gosset G. 2005. Improvement of Escherichia coli production strains by modification of the phosphoenolpyruvate:sugar phosphotransferase system. Microb Cell Factories 4:14.

3. Shiloach J, Fass R. 2005. Growing E. coli to high cell density--a historical perspective on method development. Biotechnol Adv 23:345–357.

4. Mee MT, Collins JJ, Church GM, Wang HH. 2014. Syntrophic exchange in synthetic microbial communities. Proc Natl Acad Sci 111:E2149–E2156.

5. Milo R, Phillips R. 2015. Cell Biology by the Numbers1 edition. Garland Science, New York, NY.

**(D) Statistic used for approximate Bayesian computation**

The statistics used to compare the modeling and experimental results for the approximate Bayesian computation analysis of the *E. coli* co-culture experiments are described below.

S_K_ is the ratio of the average combined growth rate of both amino acid auxotrophs in the positive control (where they were grown in the same well) divided by the growth of the lysine auxotroph in the experimental condition (where it was grown across the membrane from the isoleucine axutroph). All growth values taken at 48 hours.

S_I_ is the analogous metric for the isoleucine auxotroph.

S_K,I_ is the average of these two metrics.

The super and subscripts correspond to: I (isoleucine auxotroph), K(lysine auxotroph), L (left well), R (right well), PC (positive control, where the auxotrophs where grown in the same well), Exp (experimental condition, where the auxotrophs were grown in wells separated by a porous membrane).

$$S_{K}=\log_{10} \left( \frac{\left( B_{L,PC}^{I}\left( t=48 \right)+B_{L,PC}^{K}\left( t=48 \right)+B_{R,PC}^{I}(t=48)+B_{R,PC}^{K}(t=48) \right)/2}{B_{L,Exp}^{K}(t=48)} \right)$$

$$S_{I}=\log_{10} \left( \frac{\left( B_{L,PC}^{I}\left( t=48 \right)+B_{L,PC}^{K}\left( t=48 \right)+B_{R,PC}^{I}(t=48)+B_{R,PC}^{K}(t=48) \right)/2}{B_{R,Exp}^{I}(t=48)} \right)$$

Statistic used for equal leakage:

$S_{K,I}=\frac{S_{K}+S_{I}}{2}$_­_­
